# Evaluating Quality Parameters, the Metabolic Profile, and Other Typical Features of Selected Commercial Extra Virgin Olive Oils from Brazil

**DOI:** 10.3390/molecules25184193

**Published:** 2020-09-13

**Authors:** Aline Gabrielle Alves de Carvalho, Lucía Olmo-García, Bruna Rachel Antunes Gaspar, Alegría Carrasco-Pancorbo, Vanessa Naciuk Castelo-Branco, Alexandre Guedes Torres

**Affiliations:** 1 Laboratory of Nutritional Biochemistry and Food Science, Lipid Biochemistry and Lipidomics Laboratory, Institute of Chemistry, Federal University of Rio de Janeiro, Rio de Janeiro 21941-902, Brazil; alinegac@gmail.com (A.G.A.d.C.); brunargaspar@gmail.com (B.R.A.G.); torres@iq.ufrj.br (A.G.T.); 2 Department of Analytical Chemistry, Faculty of Sciences, University of Granada, Ave. Fuentenueva s/n, E-18071 Granada, Spain; alegriac@ugr.es; 3 Laboratory of Food Biotechnology, Fluminense Federal University, Niteroi 24230-340, RJ, Brazil; vanessanaciuk@id.uff.br

**Keywords:** extra virgin olive oil (EVOO), minor components, Brazilian EVOO, Arbequina, Koroneiki, phenolic compounds

## Abstract

The production of extra virgin olive oil (EVOO) in Brazil developed quite recently, and information on commercial Brazilian EVOO’s typical features is very scarce. In just one of the previously published works on Brazilian olive oil, the assessed samples were commercially available. In this study, a comprehensive characterization of EVOO samples acquired at local stores (at Rio de Janeiro and Rio Grande do Sul, from the two most prevalent cultivars, Arbequina and Koroneiki) was carried out considering the most relevant quality parameters, antioxidant capacity, oxidative stability, total phenolic content, fatty acid composition, and minor component metabolic profiling. The latter included: (1) the determination of individual phenolic compounds (belonging to four diverse chemical classes) and triterpenic acids by means of a powerful multi-class reversed-phase LC-MS method; (2) the quantitative profiling of tocopherols, phytosterols, and pigments by normal-phase LC-DAD/fluorescence; and (3) the quantitative appraisal of the volatile pattern of the oils by solid-phase microextraction (SPME)-gas chromatography (GC)-MS. By applying these methods, the concentrations of approximately 70 minor compounds were determined in commercial EVOOs from Brazil. To the best of our knowledge, the content of a very large number of phenolic compounds of those determined in the current report (mainly secoiridoids), the three triterpenic acids (maslinic, betulinic, and oleanolic acids), and the individual chlorophyll derivatives had not been previously evaluated in Brazilian EVOOs. The present work provides a broad picture of the compositional profile and other parameters of relevance of selected commercial Brazilian EVOOs available on local markets, describing their typicity and most particular features, some of which are known to have potential impacts on consumers’ health.

## 1. Introduction

Extra virgin olive oil (EVOO) is a well-known source of oleic acid and bioactive compounds, presenting a high potential for offering health benefits [1]. These health benefits are associated with the composition of EVOO, which is highly correlated with the olives growing region, edaphoclimatic conditions, cultivar, and ripening stage of the olives, among other factors, such as production technologies and storage conditions [2,3,4]. Therefore, EVOO from tropical and sub-tropical areas, such as Brazil, might present different chemical composition and a distinct metabolic profile in comparison with EVOO from traditional production areas [5].

Brazil is one of the world’s largest importers of olive oil, only behind the United States of America. From the 2019/20 harvest (from October 2019 to April 2020), Brazil imported more than 66,000 tons of olive oil, which is a quantity 20% higher than that of the previous harvest year [6], showing the impending relevance of the olive oil market in the country. The production of EVOO has recently emerged in Brazil, with the first batches being produced in 2008 in the Mantiqueira mountain range [7], and the first commercial EVOO reaching the market in the early 2010s.

Information on the Brazilian production of EVOO is still inconsistent and global data are not easily available, but it shows a rising tendency, with only 10,000 L of olive oil being produced in the 2013/14 harvest year, 25,000 L in 2014/15, 30,000 L in 2015/16, 105,000 L in 2016/17, 58,000 in 2017/18 (production dropped due to an adverse climate), 160,000 L in 2018/19 and, 230,000 L in the 2019/20 harvest year [8,9,10,11,12,13,14]. Up until the first semester of 2020, 77 brands of olive oil had been registered in Brazil [14], but many of these brands are sold exclusively, directly by producers or in specialized stores, limiting the availability to a restricted consumers’ market.

Currently, EVOO is produced in the South of Brazil, in the state of Rio Grande do Sul, in addition to the Mantiqueira mountain range in the country’s Southeast, in the states of Minas Gerais and São Paulo. However, climate conditions in Brazil vary considerably from the tropical North, to the sub-tropical Southeast and the temperate South. Therefore, the chemical profile in Brazilian EVOO deserves to be assessed.

Interesting studies evaluating the composition of EVOOs from Brazil have been published [7,15,16,17,18,19,20,21,22], but they are still scarce (Table S1). The first published work on the composition of virgin olive oil (VOO) produced in Brazil [7] reported the phenolic compounds (four substances), tocopherols (α-, β-, and γ-), and fatty acid composition of 17 monovarietal VOOs produced from olives farmed in the Southeast region. Later, the same group focused on characterizing the phenolic fraction (19 compounds) of twenty-five non-commercial oils from eight varieties and from three different Brazilian states by LC-MS [15]. Borges and co-workers carried out the characterization of two Arbequina VOOs produced in different regions of Brazil (Minas Gerais and Rio Grande do Sul states) and nine from Spain; they first established the physicochemical properties, oxidative stability, and fatty acid profile of the selected oils [16] and, in a following paper, evaluated the minor bioactive constituents (coenzyme Q10, tocopherols, and phenolic compounds (six phenols)) in the same oils [17]. In the same year (2017), Bruscatto et al. [18] assessed the chemical composition of four non-commercial oils produced in Southern Brazil from Arbequina, Coratina, Frantoio, and Koroneiki cultivars, determining four quality parameters, tocopherols, the total phenolic contents (spectrophotometrically), carotenoids, and chlorophylls, as well as the oxidative stability. The sensory characterization of 12 Brazilian commercial VOOs, besides the determination of other chemical parameters, was the major contribution of a complete work authored by Zago et al. [19]. Rodrigues et al. [20] tested the quality of olive oils from Southeastern Brazil (from eight cultivars), also focusing on the volatile composition and sensory characteristics of the VOOs. Very recently, one sample from each of the six different cultivars from the 2017 and 2018 harvests cultivated in Rio Grande do Sul were assessed, taking into account the quality parameters and fatty acid composition, as well as several bioactive constituents (total content of carotenoids and chlorophylls, tocopherols, and phenolic compounds (19 phenolic substances)) [21]. Topics such as the storage effects on Brazilian monovarietal EVOO have only been addressed once, by analyzing 24 experimental samples (from six varieties) from Minas Gerais [22].

The previously cited papers include stimulating and rigorous studies, but they have left gaps that deserve to be addressed. For example, in only one of the published works, the samples were commercially available, which is an important feature for giving a picture of the VOOs that are available for consumers, and therefore, that could impact consumers’ health. Additionally, the contents of very significant minor components in Brazilian EVOO have not been previously determined, and data on some secoiridoids, triterpenic acids, and individual chlorophyll derivatives are missing. Finally, a comprehensive and extensive metabolic profile of minor components has not been previously determined, which could help to improve the current information on possible biomarkers of commercial EVOOs produced in Brazil.

Minor components in VOO are comprised of phenolic and triterpenic compounds, tocopherols, sterols, volatile compounds, and pigments (chlorophylls and carotenoids), among others. The importance of some of these minor components is irrefutable; these substances can be used for the appraisal of VOO quality, purity, authenticity, and typicity [23], and they are linked to the oil’s shelf-life, sensory attributes, and several of its health-promoting effects [24]. Their determination has traditionally been intricate due to their heterogeneity and relatively low concentrations, but the development of multi-class methods has recently generated great expectations [25,26,27], as they can clearly increase the analytical throughput, providing data on a great number of substances within a single analysis.

The growing production and the emergence of new EVOO producers and brands in Brazil require a comprehensive characterization of commercial EVOOs. Therefore, the aim of the current study was to combine the use of traditional and cutting-edge methodologies for evaluating the quality parameters, antioxidant capacity, oxidative stability, total phenolic content, fatty acid composition, and metabolic profiling of minor components of ten representative Brazilian EVOOs from prevailing areas of production and cultivars. They were compared with Spanish oils from consolidated producers, as EVOOs from Spain are commonly found in Brazilian stores and represent a reference of what is commercially available for consumers in Brazil. The use of complementary liquid chromatography methodologies, i.e., reversed phase (RP)-LC-MS and normal phase (NP)-LC with diode array (DAD) and fluorescence (FLD) detectors, enhanced the coverage of the analytical determinations, providing data on individual phenolic compounds, triterpenic acids, tocopherols, phytosterols, and pigments. The overall description of the compositional profile of the oils was completed by using solid-phase microextraction (SPME)-GC-MS to determine volatiles and other methods to obtain information about additional quality features of the evaluated samples.

## 2. Results and Discussion

### 2.1. Quality Parameters, p-Anisidine Value, Antioxidant Capacity, Oxidative Stability Index, and Total Phenolic Content

EVOOs from Brazil (regardless of the cultivar) presented free acidity values, peroxide values, and specific extinction coefficients (K_232_ and K_270_) within the limits established by the International Olive Council (IOC) and the Brazilian Normative Instruction for Olive Oil and Pomace Olive Oil [28,29], with a few exceptions (Table 1). Samples from cv. Arbequina (Southeast B and Catalonia I) presented K_232_ higher than 2.50, not qualifying for EVOO or VOO categories, possibly due to the adoption of bad practices of extraction, conservation, or import times and conditions (in the case of the Spanish sample). Additionally, the *p*-anisidine value of most samples (except for Koroneiki oil from the South of Brazil, brand G) was below the maximum levels suggested by Skiera and co-workers [30]. Brazilian EVOOs showed quality parameters similar to those of Spanish samples and fulfilled the IOC requirements for the tested parameters in practically all of the cases. The antioxidant capacity did not show major differences among samples, presenting values ranging from 2.27 to 3.29 mmol of Trolox equivalent/kg. Koroneiki EVOOs were 2.8- and 2.2-fold more stable than Brazilian and Spanish Arbequina ones, respectively (Table 1: One-way ANOVA followed by Tukey’s post-hoc test, *p* ≤ 0.05). A higher oxidative stability index (OSI; h) of Koroneiki EVOOs when compared to Arbequina oils has previously been reported [18,31,32], confirming the olive cultivar influence on the oxidative stability of EVOOs.

The total phenolic content (TPC) showed a similar behavior, although the differences between Brazilian Koroneiki EVOOs and Spanish Arbequina EVOOs were of a lower magnitude (Table 1: One-way ANOVA followed by Tukey’s post-hoc test, *p* ≤ 0.05). As expected, the oxidative stability of EVOOs was strongly correlated with TPC (r = 0.8190, *p* < 0.05; Pearson’s correlation). As in the Rancimat^®^ method lipid oxidation is accelerated, samples presenting higher contents of antioxidants such as phenolic compounds are more likely to show longer induction times. 

Additionally, cv. Koroneiki samples from the Southeast of Brazil, where growing fields are located at higher altitudes, presented longer induction times and higher TPC, when compared to samples from olives cultivated in the South of Brazil. Similar behavior has already been described by other authors, who reported correlations of higher TPC in olives cultivated at higher altitudes for Brazilian and Spanish [17] and Tunisian EVOOs [4]. This observation may be explained by the fact that olives grown at higher altitudes generally exhibit slower rates of maturation, thus delaying the effects of ripening on decreasing the contents of natural antioxidants, including phenolic compounds [33].

### 2.2. Fatty Acid Composition in EVOO Samples

Fatty acid profiles were determined by GC with a flame ionization detector (FID) and were quite consistent in all of the samples, exhibiting the expected values for EVOO, especially concerning the oleic acid content [28,29]. Koroneiki EVOOs displayed a higher monounsaturated to polyunsaturated ratio (M:P_ratio_) than Arbequina EVOOs, regardless of the country of production (Table 2), as previously observed in EVOOs from Tunisia [32,34]. The olive cultivar has been reported as one of the main factors influencing the fatty acid profile, together with the stage of maturation, geographical location, and climate [2,3]. These data highlight the interesting nutritional properties of cv. Koroneiki EVOOs, as the relatively high M:P_ratio_ is indicative of a high-quality dietary lipid source with potential health benefits related to the prevention of chronic diseases, such as diabetes and cardiovascular disease [35,36].

### 2.3. Minor Components

Minor components were comprehensively evaluated in the commercial EVOO samples under study by means of three methods: RP-LC-MS was used to determine the most polar EVOO fraction; NP-LC-DAD/FLD was used to study less polar metabolites; and SPME-GC-MS was employed to assess the EVOO volatile and semi-volatile profile. In total, 62 minor components were identified and quantified in EVOO by two complementary LC methods and eight volatiles were determined by GC. In other words, the contents of 70 substances were established, as follows: Phenolic compounds (simple phenols, phenolic acids, and related compounds (ten); secoiridoids and derivatives (twenty-seven); flavonoids (four); and lignans (three)); triterpenic compounds (three); free fatty acids (three); tocopherols (four); pigments (six); phytosterols (two); and eight compounds from the volatile fraction. In the coming sections, the quantitative results achieved by using each analytical platform will be discussed. 

#### 2.3.1. Phenolic Compounds, Pentacyclic Triterpenes, and Free Fatty Acids

Overall, in the present study, secoiridoids were the most prevalent sub-class of phenolic compounds, followed by lignans, simple phenols and similar substances, and flavonoids (Table S2a), which is in accordance with previous reports [37].

**Figure 1:**
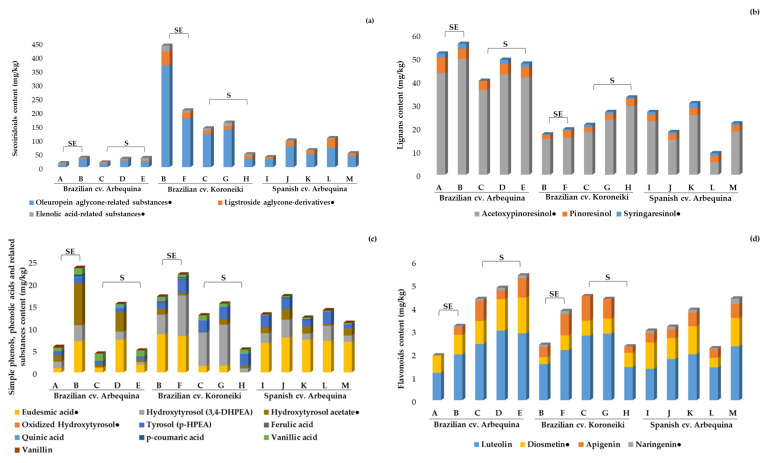

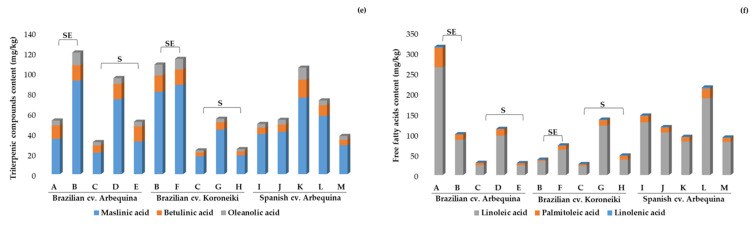
Minor component profiles (mg/kg) of the studied EVOOs determined by reversed phase (RP)-LC-MS (sample identification in Table 3): (**a**) Secoiridoids; (**b**) lignans; (**c**) simple phenols, phenolic acids, and related substances; (**d**) flavonoids; (**e**) triterpenic compounds; and (**f**) free fatty acids. SE, Samples from the Southeast. S, Samples from the South. ^●^ Compounds quantified in mg of homologous substance/kg, as shown in Table S5a.

Before discussing the quantitative results and for the sake of clarity, it should be mentioned that the direct comparison of our outcomes with other findings previously published in the literature must be treated with caution because of two main factors: (i) Many of the compounds evaluated in the current study have not been assessed by previous works on Brazilian oils, and (ii) whenever commercial standards were unavailable, quantitative analysis was based on the calibration curves of analogous substances. Therefore, there is a chance that, in each work, different analogous compounds were used for calibration, rendering different responses. In those cases, the quantification is relative, so comparisons should be cautiously made. Total secoiridoids contents were between 14.5 and 33.4 mg/kg (quantified based on the oleuropein calibration curve) in Brazilian Arbequina EVOOs; between 36.2 and 105.0 mg/kg in Spanish Arbequina oils; and showed a wider variation, from 46.6 to 439.0 mg/kg, in Koroneiki samples. In all samples, secoiridoids represented a considerable percentage of the total contents of phenolic compounds. Fourteen compounds (plus thirteen isomers) from this class were identified and quantified in the samples, with elenolic acid, oleuropein aglycone, and ligstroside aglycone being some of the most abundant (Figure 1a; Table S2a). 

Brazilian cv. Koroneiki EVOOs from the Southeast showed the highest concentrations of total secoiridoids (439 and 205 mg/kg; mostly oleuropein aglycone) among the 15 analyzed samples, which makes these oils interesting sources of such compounds that present potential anti-inflammatory and antioxidant effects [37]. These contents were, on average, more than 6.2-fold and 2.9-fold higher, respectively, than those found in the Spanish cv. Arbequina, and more than 16.8-fold and 7.8-fold higher, respectively, than those found in the Brazilian cv. Arbequina. Previous reports on EVOO from Brazil found fewer compounds belonging to this class. For instance, Zago and co-workers quantified four secoiridoids and their total contents were between 6.83 and 53.1 mg/kg (quantified based on the gallic acid calibration curve) for all of the varieties they tested [19]. Crizel and her team determined eight secoiridoids and their contents in Arbequina and Koroneiki EVOOs varied between 89.5 and 111.2 and 177.8 and 185.6 mg/kg (using oleuropein for the quantification), respectively, in consecutive harvest years (2017 and 2018) [21]. Ballus et al. [15] evaluated the content of nine complex phenols (based on the oleuropein calibration curve) in EVOOs from Brazil and found contents of total secoiridoids varying from about 0.4 to 59.1 and from 13.3 to 133.5 mg/kg in cv. Arbequina and Koroneiki oils, respectively. Although the differences found between the data in the present work and those previously published might be due to methodological issues, it is also likely that at least some of these contrasting values were related to geographical and edaphoclimatic factors, as well as the adopted technological processes. 

The glycosylated forms of secoiridoids are not often detected in EVOO due to their high solubility in water and low solubility in oil. Nevertheless, the aglycones that are formed by enzymatic hydrolysis during oil extraction represent one of the major classes of phenolic compounds in EVOO [1]. As previously reported, several isomers of the aldehydic form of oleuropein and ligstroside aglycones, decarboxymethyl oleuropein aglycone, and decarboxymethyl ligstroside aglycone can be artificially formed during sample preparation or analysis, either by interaction with methanol (and probably water and/or their mixtures) during extraction or with the silica-based stationary phase in a chromatographic column [38,39]. To circumvent this issue, we adopted a previously described procedure [40,41,42], in which the use of methanol was eluded and the quantitative analysis was carried out by calculating each isomer’s content individually, in order to prevent underestimating the total content of these compounds. Lignans were the major group of phenolic compounds in cv. Arbequina oils from Brazil, with contents varying from 40.2 to 56.0 mg/kg, which were, on average, 2.3-fold and 2-fold higher than in cv. Koroneiki EVOOs and Spanish cv. Arbequina oils, respectively. Acetoxypinoresinol was the most abundant phenolic compound in all samples, followed by pinoresinol and syringaresinol (Figure 1b; Table S2a). Arbequina EVOOs from Brazil showed contents of acetoxypinoresinol varying from 36.2 to 49.5 mg/kg, which were almost 2.5-fold higher than the Spanish ones and 2-fold higher than cv. Koroneiki EVOOs. Higher contents of acetoxypinoresinol and pinoresinol in cv. Arbequina EVOO when compared to oils from cv. Koroneiki and cv. Picual have been previously described. Besides variation among olive cultivars, olive oils might differ in these compounds’ contents due to fruits’ ripening stage, the extraction technologies used, and the storage practices adopted [15,21,43]. Brazilian Arbequina EVOO seems to be a richer source of lignans, when compared to Brazilian Koroneiki and Spanish EVOOs. Therefore, the consumption of Brazilian Arbequina EVOOs would be preferable for increasing the lignans intake, motivated by the fact that these compounds have positive effects on human health, such as chemoprotective and anti-inflammatory actions [44].

The amounts of simple phenols, phenolic acids, and related substances varied widely in the present work. The total contents (mg/kg) of these compounds in cv. Arbequina EVOOs varied from 4.23 to 23.5 in Brazilian and from 5.12 to 22.0 in Spanish oils, and from 11.1 to 17.1 in cv. Koroneiki EVOOs. In general, simple phenols and related analytes represented approx. 3.6 to 20% of the total phenolic compounds in all samples (Figure 1c; Table S2a). Hydroxytyrosol and tyrosol are two well-known bioactive compounds, which present antioxidant and anti-inflammatory effects [37], and are at least partly responsible for the beneficial effects of olive oil consumption. The hydroxytyrosol content varied from trace levels to representing a remarkable percentage of the overall simple phenols, phenolic acid, and related substances content in some samples. The highest concentration levels of this compound were found in cv. Koroneiki EVOOs, which were, on average, three times and twice as high as those in cv. Arbequina from Brazil and Spain, respectively. Brazilian cv. Arbequina EVOOs presented some of the lowest contents of hydroxytyrosol, with it being below the limit of detection in a sample from the South region (Brand C). Arbequina oils also exhibited low contents of tyrosol that varied from 0.72 to 2.88 mg/kg. These levels were generally in the same range as those previously reported in both commercial and “Abencor” EVOOs produced in Brazil from cv. Arbequina and cv. Koroneiki [15,19]. Ballus and his team reported concentrations of tyrosol and hydroxytyrosol ranging from traces of both compounds to 9.4 mg/kg of tyrosol and to 14 mg/kg of hydroxytyrosol, respectively, in Arbequina EVOOs from successive harvests between the years 2010 and 2012 [7,15].

Borges et al. [17] determined hydroxytyrosol in Arbequina EVOOs from Brazil and Spain; small quantities of this substance were found in Arbequina EVOO samples from the South (1.05 mg/kg) and Southeast (0.092 mg/kg) of Brazil. Moreover, Crizel and co-workers found that the concentrations of hydroxytyrosol were within the range of 0.45–1.21 and 0.45–1.89 mg/kg for “Abencor” Arbequina and Koroneiki oils produced in Southern Brazil in 2017 and 2018, respectively [21]. Moreover, they showed very high concentration levels of tyrosol in the same work, varying from 45.0 to 330.6 mg/kg in Arbequina VOO and 38.5 to 101.3 mg/kg for Koroneiki oils, respectively [21]. 

Establishing the content of these substances in a reliable way is of great importance, as these compounds are regarded as responsible for part of EVOO’s bioactivity, and even in small quantities, these metabolites are known for being powerful antioxidants [45].

As expected [1], flavonoids were found in relatively small amounts in EVOOs in the present study, with luteolin being the most abundant one in every sample, followed by diosmetin, apigenin, and naringenin (Figure 1d; Table S2a). The intake of flavonoids, even in small amounts, has been reported to reduce the risk of death from cardiovascular disease [46]. Luteolin contents did not vary substantially among the samples analyzed herein (from 1.18 to 3.00 mg/kg), irrespective of the origin and olive variety. The concentrations found in the present work were similar to those previously reported in EVOO from Brazil [19,21]. Other interesting studies on EVOO from Brazil have reported the following concentration values for luteolin: 2.4 to 11 mg/kg for cv. Arbequina and 4 to 10 mg/kg for cv. Koroneiki [15], and 1.48 mg/kg in Arbequina EVOO samples from the South and 0.09 mg/kg in samples from the Southeast regions of Brazil [17].

In the present study, three pentacyclic triterpenes (maslinic, betulinic, and oleanolic acids) were identified and quantified (Figure 1e; Table S2a), with the first being the predominant compound of this class in all samples. To the best of our knowledge, there are no previously published works reporting the concentrations of triterpenic acids in Brazilian EVOOs. Among the samples from Brazil, most EVOOs from the Southeast presented higher contents of such compounds (except for cv. Arbequina brand A, with 52.9 mg total triterpenic acids per kg), when compared to samples from the South. Contents of maslinic acid in EVOOs from Brazil would be equivalent to those in the group with very high or high contents of pentacyclic triterpenes among the samples from the World Olive Germplasm Bank Collection of Cordoba (Spain) established by Allouche et al. [47]. These authors carried out a comprehensive study evaluating the triterpenic dialcohol and acid composition of oils from 40 cultivars, and divided them into five groups according to their content (as betulinic acid equivalents). They primarily assigned the differences in triterpenoids content to genetic factors. However, when oleanolic acid is considered, Brazilian EVOOs would be placed in the low content group. Oleanolic acid concentrations varied from 2.03 to 12.6 mg/kg in a Koroneiki sample and in one Arbequina EVOO from Brazil, respectively. 

The relevance of determining pentacyclic triterpenes in EVOO lies in their potential benefits for human health, especially in inflammatory and arthritic diseases that might be prevented or attenuated by maslinic acid; besides, oleanolic acid has exhibited bioactivity against diabetes and metabolic syndrome [48]. Free palmitoleic, linoleic, and linolenic acids were also determined with the multiclass method used herein (Figure 1f; Table S2a); that is why we included the quantitative information of these three substances (directly determined from their respective calibration curves) in the current section, even though the fatty acid composition (%) of the oils was shown in Section 2.2, according to the official method. These data provide different information, as, for the analysis of the fatty acid composition by GC-FID, fatty acids of virtually all of the lipid classes are methylated and determined, as opposed to the free fatty acids analyzed in the multiclass method.

#### 2.3.2. Tocopherols, Sterols, and Pigments

NP-LC coupled to two different detectors was also used in this study to complete the thorough information provided by the RP-LC-MS method. Therefore, the tocopherols and pigments profiles were characterized by NP-LC, which also procured the contents of lupeol and total free sterols.

**Figure 2:**
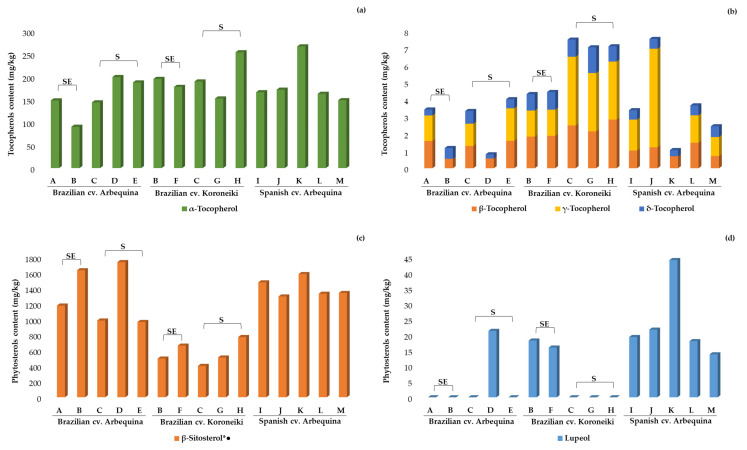

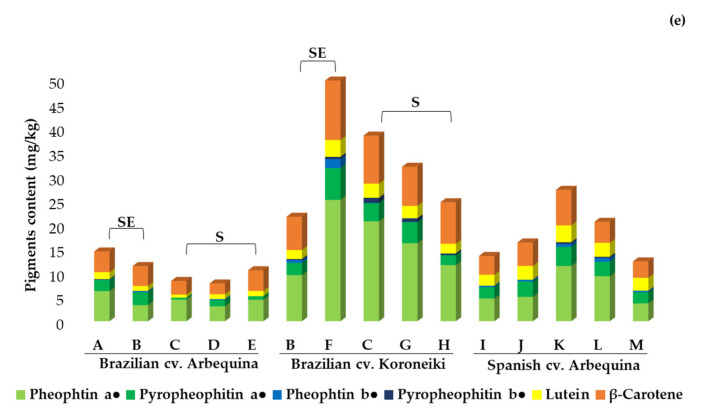
Tocopherols, phytosterols, and pigments profiles (mg/kg) of the studied EVOOs determined by NP-LC-DAD/FLD (sample identification in Table 3): (**a**) α-tocopherol; (**b**) ββ-, γ-, and δ-tocopherols; (**c**) phytosterols: *β-sitosterol, coeluted with other free sterols; (**d**) lupeol; and (**e**) pigments. SE, Oils from the Southeast. S, Oils from the South. ^●^ Compounds quantified in mg of homologous substance/kg, as shown in Table S5b.

The tocopherols profile followed the expected pattern for this type of oil in all of the samples. αα-tocopherol was the most prevalent one in every oil, accounting for over 90% of the total tocopherols content. This substance is well-known for its ability to act as a hydroperoxyl radical scavenger and plays an important role in protecting organisms against oxidative damage [49]. αα-tocopherol (Figure 2a; Table S2b) was accompanied by low contents of β-, γ-, and δ-tocopherols (Figure 2b; Table S2b), in agreement with previous reports on EVOO from Brazil and Spain [7,17,18,21]. Concerning phytosterols, it is necessary to mention that only free sterols were assessed in the present work, because the developed method did not include a saponification step. Moreover, the applied NP-LC separation essentially intended to provide an exhaustive profiling of tocopherols and pigments, rather than characterize the sterols profile. In any case, the contents of free sterols were highly variable among Brazilian EVOOs (much more so than in the Spanish samples). This high variability seemed to be related to both the production region (South vs. Southeast) and olive cultivar (Figure 2c; Table S2b). This is the first study reporting the contents of sterols in EVOO from Brazil, so we should analyze a wider number of samples and compare these results with those achieved by GC after saponification for the purpose of drawing meaningful conclusions. However, we should not discard the hypothesis that the variation in oil extraction technologies adopted by producers could, at least partially, explain the high variation observed in EVOOs from Brazil, because sterol esters could have been hydrolyzed during olive fruits processing. This assumption deserves proper confirmation in future studies specifically designed to test it. In addition, it reinforces the need for more research on EVOO from Brazil. 

The pigments profile determines EVOO’s color and might have an influence on the oil stability. To the best of our knowledge, the detailed profiling of chlorophyll-derived pigments and carotenoids has not been previously characterized in EVOOs from Brazil. Among pigments, pheophytin *a*, which is a chlorophyll derivative, and β-carotene were found at higher concentrations (Figure 2e; Table S2b). Both pigments’ classes have the potential to act as antioxidants in EVOO, if they are protected from light; otherwise, they may act as pro-oxidants [50]. Overall, the pheophytin *a* content in Brazilian EVOOs accounted for 53–92% of the total chlorophyll derivatives, exhibiting concentrations from 3.10 to 25.1 mg/kg, and its contents in cv. Koroneiki samples were 4-fold and 2-fold higher than those of cv. Arbequina from Brazil and Spain, respectively. Previous studies of Brazilian EVOO have reported very variable total chlorophylls contents (mg/kg), ranging as follows: From 1.39 to 1.73 [16]; between 0.1 and 0.9 in Arbequina oils and 0.5 and 2.6 in Koroneiki oils [21]; from 14.06 to 59.93 [19]; and 0.84 and 0.90 in Arbequina and Koroneiki oils, respectively [18]. The high proportion of pheophytin *a* in commercial samples can account for the rapid conversion of chlorophylls in freshly extracted EVOO to more stable derivatives, such as pheophytin, and subsequently to pyropheophytin, due to the loss of the central magnesium and the carboxy-methyl group, respectively [51]. It has been previously described that the storage of EVOO in the dark at 15 °C promoted the transformation of chlorophylls into their derivatives in about 2 to 3 months, possibly by the action of olives’ natural acids released during the milling and paste beating steps of EVOO processing [52]. Koroneiki EVOOs from Brazil also presented the highest contents of β-carotene (6.90–12.3 mg/kg, quantified in terms of its own pure standard), which were 2.5-fold and 2-fold higher (on average) than the contents in cv. Arbequina EVOOs from Brazil and Spain, respectively. In contrast, lutein contents were similar in cv. Koroneiki and Spanish cv. Arbequina EVOOs, which were approximately 3-fold higher than in cv. Arbequina from Brazil (0.62–1.45 mg/kg). The total carotenoids in previously published works have been found to have the following contents (mg/kg) in Brazilian oils: 2.17–3.85 in Arbequina oils [16]; 3.8–12.8 in Arbequina and 6.3–12.7 in Koroneiki oils [21]; 10.69–26.18 in several olives’ varieties [19]; and 3.97 in Arbequina and 4.75 in Koroneiki oils [18]. 

Besides the obvious influence of processing and storage, the observed variation in the contents of pigments could be attributed to genetic factors [53], which could explain the differences between cv. Koroneiki and cv. Arbequina. β-carotene can positively impact human health, either directly or by its pro Vitamin A activity, via conversion to retinoids [51].

#### 2.3.3. Volatile Composition

**Figure 3:**
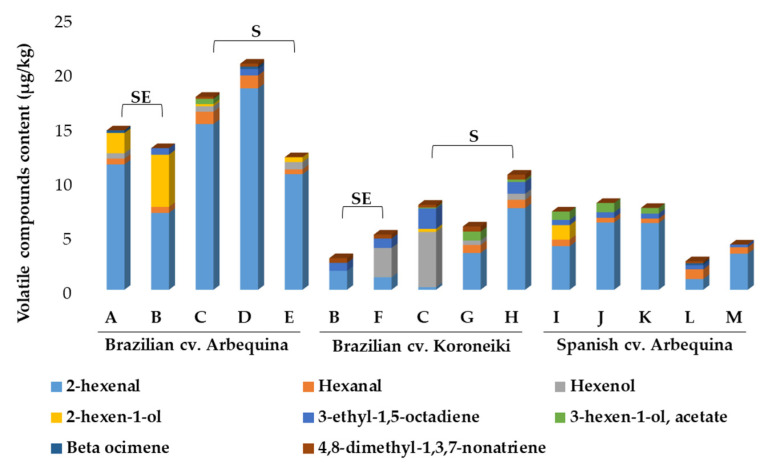
Volatile and semi-volatile compounds profile (µg/g) of the studied EVOOs determined by solid-phase microextraction (SPME)-GC-MS (sample identification in Table 3). SE, Samples from the Southeast. S, Samples from the South.

Eight volatile compounds belonging to the chemical classes of aldehydes, alcohols, hydrocarbons, and esters were determined in EVOO samples by SPME-GC-MS (Figure 3; Table S3). As expected, 2-hexenal was the major volatile compound in all samples (reaching a value of 18.5 µg/g), except for two EVOOs from cv. Koroneiki. Aldehydes are often reported as major volatile compounds in EVOO, with 2-hexenal usually being the most abundant in oils from Brazil [19,20] and other origins [54]. 2-hexenal is associated with a green/apple-like odor, with an odoriferous threshold varying from 420 to 1125 μg/kg oil [55,56]. This compound is possibly the main one responsible for the characteristic fresh and green aroma of EVOO. Hexanal was detected in most of the EVOO samples analyzed herein, with concentrations varying from 0.41 to 1.18 µg/g, and even though its mass spectrometry similarity index (SI = 61%) was lower than the one found for other compounds, the diagnostic fragments at *m*/*z* 44, 56, 72, and 82 match the signal profile expected for this compound; the signals at *m*/*z* 56, 72, and 82 relative to those of *m*/*z* 44 were (mean ± SD) 0.93 ± 0.05, 0.32 ± 0.02, and 0.27 ± 0.03, respectively, which were similar to those of a refence spectrum (0.83, 0.21, and 0.15), even though the base peak (*m*/*z* 41) was not the same as in the reference spectrum (*m*/*z* 44). Furthermore, the experimental linear retention index was close to previously published data (Error_LRI_ < 0.5%). Taken together, these data meet the criteria for tentative peak assignment. This aldehyde is commonly found in low contents in EVOO as it can be formed in the olives by the enzymatic oxidation (by lipoxygenases) of polyunsaturated fatty acids [57], and as long as it is not extensively accumulated in the fresh oil, it will probably not have a negative impact on the oil’s quality. It gives the EVOO a green flavor when present in relatively small amounts, but is largely regarded as responsible for vegetable oils’ rancid flavor (when present at high concentrations), as it can be produced by the oxidative degradation of linoleic acid. 

Two six-carbon unsaturated alcohols were identified. 3-hexen-1-ol, which is linked to fruity odor notes [58], was the major compound in two of the cv. Koroneiki EVOOs and was not detectable in any of the cv. Arbequina samples from Spain. 2-hexen-1-ol was mainly found in Brazilian Arbequina oils, with values varying from 0.20 to 4.79 µg/g, and was quantifiable in only one of the Arbequina samples from Spain (Catalonia brand I) and one Koroneiki oil (South brand C). Beta ocimene, which is a monoterpene hydrocarbon, was only detected in three samples (0.18–0.24 µg/g), and was not found in EVOOs from cv. Koroneiki olives. This compound has been previously described in VOO and EVOO [54,59,60] and was reported to contribute with sweet and herbal aroma notes [61]. 

The single identified ester was 3-hexen-1-ol acetate, which was detected in samples from the South of Brazil (except for samples D and E from cv. Arbequina) and in three of the five Spanish samples. This ester is also derived from the lipoxygenase pathway, where acetyl-CoA derivatives suffer from the action of alcohol acyltransferase to form acetate esters, which contribute to aroma notes of banana and walnut husk [54,62]. 

## 3. Methods and Materials

### 3.1. Materials

All reagents and chemicals used in this study were of analytical grade, and solvents used for chromatography were, appropriately, HPLC or LC-MS grade. Methanol, acetonitrile, acetic acid, *n*-hexane, and isopropanol were used to prepare mobile phases (Sigma-Aldrich^®^, St. Louis, MO, USA; Merck, Darmstadt, Germany; Tedia, Fairfield, OH, USA). Ethanol (EtOH), acetone, and chloroform, used for extractions and the assessment of quality parameters, were obtained from J.T. Baker (Deventer, The Netherlands). Ultrapure water (Milli-Q system, Millipore, Bedford, MA, USA) was freshly obtained before use. Analytical grade standards used for quantification purposes, including phenolic compounds (quinic, vanillic, *p*-coumaric and ferulic acids, hydroxytyrosol, tyrosol, vanillin, luteolin, and apigenin), pentacyclic triterpenes (maslinic, betulinic, and oleanolic acids), tocopherols (α-, β-, γ-, and δ-tocopherols), free fatty acids (palmitoleic, linoleic, and linolenic acids), chlorophylls *a* and *b*, lutein, β-carotene, and lupeol were acquired from Sigma-Aldrich^®^. In addition, pinoresinol was purchased from Arbo Nova (Turku, Finland), oleuropein from Extrasynthese (Lyon, France), and β-sitosterol from Coompo Research Chemicals (Wuhan, China). A standard mixture containing C7-C30 saturated alkane standards was purchased from Supelco (Sigma-Aldrich^®^, St. Louis, MO, USA).

### 3.2. Extra Virgin Olive Oil Samples

Fifteen commercial samples of monovarietal EVOO (three bottles from each) from Brazil and Spain were obtained at local shops, websites of specialized stores, and producers in Rio de Janeiro and Rio Grande do Sul. Ten samples of monovarietal commercial Brazilian EVOO from the most prevalent cultivars Arbequina and Koroneiki were selected (four from the Southeast and six from the South of Brazil) from the 2015/2016 harvest (Table 3). These ten samples represent 53% of the monovarietals that were available by 2017 [14,63]. Five commercial Arbequina EVOOs from Spain (Catalonia) were also acquired in Brazil and used as representative samples based on two criteria: (a) The profile of samples from this olive variety and geographical origin, which have been extensively described in previous studies [16,17,25,33], and (b) Spain is the largest exporter (52% of world’s exportations from European Union) of this product [64], and its oils are largely found in Brazilian markets, providing an overview of EVOOs generally available for consumers in this country. Table 3 shows samples’ country of origin, cultivar, region of production, and commercial brand letter code, as well as their production locations’ latitude, longitude, and altitude, as stated by their respective producers.

Three bottles of 250 mL of each Brazilian EVOO from the same production batch were homogenized to compose one sample. The same procedure was applied for Spanish EVOO using two 500 mL bottles. Extra virgin olive oils’ bottles were opened within 6 months after being bottled up, and once opened, all samples were placed in polyethylene bottles, purged with nitrogen, and stored whilst being covered with aluminum foil at −18 °C until analysis. 

### 3.3. Quality Parameters, p-Anisidine Values, Antioxidant Capacity, Oxidative Stability Index, and Total Phenolic Content

Free acidity, peroxide, and *p*-anisidine values were determined by official methods (Methods Cd 3d-63, Cd 8b-90, and Cd 18-90, respectively) [65]. The specific extinction coefficients K_232_ and K_270_ were determined spectrophotometrically (Method Ch 5-91) [65]. The antioxidant capacity was determined by the Trolox equivalent antioxidant capacity (TEAC) assay [66] and the oxidative stability index was determined with Rancimat^®^ equipment (743 Rancimat^®^; Metrohm^®^, Herisau, Switzerland) (20 L/h; 110 ± 1.5 °C). The total phenolic content was determined by the Folin-Ciocalteu assay method with adaptations [67], after methanolic extraction [68].

### 3.4. Fatty Acid Composition by GC-FID

The EVOO samples were methylated [69] and fatty acid methyl esters (FAMEs) were analyzed by gas chromatography (GC-2010; Shimadzu^®^, Kyoto, Japan). A polyethylene glycol capillary column (Omegawax 320^®^; 30 m × 0.32 mm, 0.25 μm; Sigma-Aldrich^®^, Sao Paulo, Brazil) was used, with the column oven temperature programmed to start at 160 °C for 2 min, followed by a temperature gradient of 2.5 °C/min up to 190 °C, held for 5 min, which was then followed by a gradient of 3.5 °C/min up to 220 °C, held for 15 min. Helium was used as carrier gas (linear velocity of 25.0 cm/s), and the injector was operated at 260 °C with a split injection at a 1:20 split ratio. FID was operated at 280 °C, with a hydrogen flow rate of 40 mL/min and air flow rate of 400 mL/min. A commercial mixture of FAME standards (Supelco^®^ 37 Component FAME Mix; Sigma-Aldrich^®^, Sao Paulo, Brazil) was used to identify the samples’ FAMEs by comparison to their relative retention times. The software Lab Solutions GC (version 2.30.00, 2004; Shimadzu^®^, Kyoto, Japan) was used for GC data analysis. Fatty acid contents were determined by internal normalization, after area correction by theoretical correction factors that also convert the content of FAME to the content of fatty acid [70]. The analyses were conducted in triplicate, and the results were expressed in g/100 g of total fatty acids. 

### 3.5. Minor Component Profiling of EVOO Samples

#### 3.5.1. Analysis of Minor Components in EVOO by RP-LC-MS

Phenolic compounds, pentacyclic triterpenes, and free fatty acids were simultaneously determined by RP-LC-MS, preceded by unselective liquid-liquid extraction (protocol adapted from [27]). The EVOO polar fraction was extracted in duplicate by adding 10.0 mL of EtOH:H_2_O (60:40, *v*/*v*) to 1.0 g of oil, followed by vortex agitation for 3 min and centrifugation (8000 rpm, 5 min, room temperature; Sigma 2-16P centrifuge, rotor 12181; Osterode, Germany); the polar phase was collected and the oil-phase was re-extracted twice with 10.0 mL of EtOH:H_2_O (80:20, *v*/*v*). The extracts were combined, evaporated in a rotary evaporator (Rotavapor^®^ R-215, Büchi^®^, Toronto, Canada), and reconstituted in 1.0 mL of EtOH:H_2_O (80:20, *v*/*v*). 

An LC system (Agilent 1260 LC system; Agilent Technologies, Waldbronn, Germany) coupled to an ion-trap (IT) MS (Bruker Daltonics Esquire 2000TM; Bruker Daltonik, Bremen, Germany) with an electrospray ionization (ESI) source was used to determine the minor components profile. Compound separation was carried out by using a RP column (Zorbax Extend C18; 4.6 × 100 mm, 1.8 µm particle size; Agilent Technologies) at 40° C. 10 µL was set as the optimum autosampler volume injection. Analytes were eluted with a mobile-phase gradient of acidified water (0.5 % acetic acid, *v*/*v*) (A) and acidified acetonitrile (0.5 % acetic acid, *v*/*v*) (B), at a 1.0 mL/min flow rate, with a total run time of 27.5 min, as follows: 0 to 2 min, 10–25% B; 2 to 16 min, 25–60% B; 16 to 18 min, 60–80% B; 18 to 23 min, 80–100% B (kept for 3 min); and returning from 100 to 10% B in 1.5 min, followed by a 2.5 min post run re-equilibration. A post-column split (1:4 ratio) was used to reduce the flow being delivered into the MS. ESI conditions were as follows: The nebulizer pressure was set at 30 psi, drying gas temperature at 300 °C, and drying gas flow at 9 L/min. The MS detector was operated in negative mode using two different capillary voltage segments: +3200 V between 1 and 17 min, and +3500 V from 17 to the end of the run. The skimmer, octopole, and lense voltages were tuned considering the average mass which was set as the target mass value for each segment. Spectra were recorded in full scan mode (50–1000 *m*/*z*). Additionally, a high-resolution MS detector was used to obtain accurate *m*/*z* signals of the detected compounds, in order to confirm their identity. To that end, some representative sample pools were analyzed with an Acquity UPLC™ (ultra-performance liquid chromatography) H-Class system coupled to a QTOF SYNAPT G2 MS (Waters, Manchester, UK) through an ESI interface. Compound identification (Table S4a) was achieved by comparisons with commercially available standards’ MS spectra and retention times, molecular formulae calculated from the exact *m*/*z*, and the expected elution order. Analyte quantification (Table S5a) was achieved by interpolating area values obtained from extracted ion chromatograms (EICs), on the external calibration curves of each standard compound or the one with the closest chemical structure available. The content of compounds lacking a pure standard was thus expressed as mg of an analogous substance/kg of olive oil. Representative EICs of EVOO samples analyzed by RP-LC-MS are available in Figure S1a. The software Data Analysis 4.0 (Bruker Daltonik) was used for LC-MS data analysis. 

#### 3.5.2. Analysis of Tocopherols, Phytosterols, Chlorophylls, and Carotenoids by NP-LC-DAD/FLD

Tocopherols, chlorophylls, carotenoids, and phytosterols were simultaneously determined by NP-LC-DAD/FLD. EVOO was dissolved, in duplicate, in hexane:isopropanol (99:1, *v*/*v*); vortex-homogenized; and filtered through a 0.45 µm pore diameter PTFE filter (Agela Technologies; Wilmington, DE, USA). A normal phase column (ZORBAX Rx-Sil, 4.6 × 100 mm, 1.8 µm, 600 bar; Agilent Technologies^®^, Santa Clara, CA, USA) was used to separate the compounds with a mobile-phase gradient of hexane (A) and isopropanol (B), at a 1.0 mL·min^−1^ flow rate, as follows: 1% of B (kept for 12 min); from 12 to 14 min, 1–10% of B (kept for 1 min); and 15–16 min, 10–1% of B. The total run time was 16 min, with a 10 min post run for column equilibration. FLD was used to detect tocopherols and to confirm the identity of chlorophyll peaks (tocopherols: λ_exc_ = 295 nm and λ_em_ = 330 nm, from 0.01 to 4.3 min; chlorophylls: λ_exc_ = 430 nm and λ_em_ = 660 nm, starting at 4.31 min). Photomultiplier tube (PMT) gain was modified during the analysis, starting at 10; it was changed to 12 at 2.2 min, followed by a change to 15 at 4.3 min. 

DAD was used to detect chlorophylls and their derivates (pheophytin *a* and pyropheophitin *a*: λ = 409 nm; chlorophyll *a*, pheophytin *b,* and pyropheophytin *b*: λ = 430 nm; chlorophyll *b*: λ = 454 nm), carotenoids (lutein: λ = 446 nm; β-carotene: λ = 454 nm), and free phytosterols (λ = 210 nm). Peak assignment was achieved based on the retention time, UV/Vis spectra, and peak shape of the standards; fluorescence excitation and emission spectra were also used for the confirmation of tocopherol and chlorophyll identities (Table S4b). Calibration curves containing 9 to 12 levels of commercial or in-house prepared standards (see below) were used for quantitative analysis, as detailed in Table S5b. All analyses were conducted in duplicate and results were expressed in mg/kg. Representative chromatograms of EVOO samples analyzed by NP-LC-DAD/FLD are available in Figure S1b. The software ChemStation for LC 3D systems (Rev. B.04.03 [16]) (Agilent Technologies) was used for instrument control and data analysis.

Pheophytins were obtained from the degradation of chlorophylls extracted from spinach [71,72], and pyropheophytins were obtained by heating the solutions of pheophytins in a mineral oil bath at 110 °C for 18 h. Prepared standards had their identities and concentrations determined spectrophotometrically (Varian Cary 50 Conc Spectrophotometer; Agilent Technologies^®^, USA). 

#### 3.5.3. Analysis of Volatile and Semi-Volatile Compounds by SPME-GC-MS

Volatile and semi-volatile compounds were extracted from the EVOO by SPME and analyzed by GC-MS [73,74,75]. Briefly, 1 g of EVOO was added to a headspace vial, with 20 μL of bromebenzene (0.1 mg/mL) and a stirring bar. The headspace vial was sealed and taken to a glycerol bath at 40 °C for 30 min, with constant magnetic agitation, followed by exposure of the manual SPME syringe fiber (Divinylbenzene/Carboxen/Polydimethylsiloxane; Supelco^®^, Bellefonte, PA, USA) to the sealed headspace vial for 10 min. Volatile compounds were desorbed by exposing the SPME syringe fiber into the injector port at 260 °C (split ratio 5:1) for 3 min and were eluted into a fused silica 5% phenyl/95% methylpolysiloxane capillary column (30 m × 0.25 mm ID, 0.25 μm; HP-5MS; Agilent Technologies, USA), with an injector pressure (He) of 49 Kpa and He linear velocity of 25.0 cm/s in a GC coupled to a single quadrupole mass spectrometer (7890A GC System coupled to 5975C MS detector, Agilent Technologies, USA). The column oven temperature was programed to start at 30 °C, held for 10 min, increased at 3 °C/min up to 200 °C, and held for 10 min before cooling back to the initial temperature. The electron impact source was operated at 70 *e*V and full scan spectra were recorded in the mass range of 40–500 *m*/*z* at 3.2 scan/s. The interface and ion source temperatures were set at 260 °C. To calculate the linear retention index (LRI), a mixture of C7–C30 hydrocarbons was run under the same conditions as samples. Volatile compounds were tentatively identified by a comparison of mass spectra with those of the National Institute of Standards (NIST) mass spectra library and similarity indices (SI) calculated by the instrument’s software (MSD ChemStation v. F.01.00.1903, Agilent Technologies, USA), and also by comparing LRI values with previously published data, considering SI > 60% and Error_LRI_ < 0.5%, or SI > 80% as criteria for tentative peak assignment. Table S6 shows the aroma notes and identification conditions of EVOOs from Brazil and Spain. Analyses were conducted in triplicate and results were expressed in µg/g.

### 3.6. Statistical Analysis

Results were expressed as the mean ± standard deviation. One-way ANOVA followed by Tukey’s post-hoc test was used to compare average values, employing the GraphPad Prism software (version 8.0.1, GraphPad Software, San Diego, CA, USA). For all of the analyses, *p*-values ≤ 0.05 were considered significant.

## 4. Conclusions

The present work contributes to drawing a picture of Brazilian commercial monovarietal Arbequina and Koroneiki olive oils, showing that the chemical quality attributes of these Brazilian oils are comparable to those found in some of the most consistent production regions in the world, in terms of the quality indices, antioxidant capacity, oxidative stability, total phenolic content, fatty acid composition, and metabolic profiling of minor components, which are known to have an impact on consumers’ health. The combination of several chromatographic methodologies and other analytical approaches have made it possible to obtain quantitative information about 70 minor components of great significance and seven further quality parameters. The content of a large number of phenolic compounds, the three triterpenic acids, and the individual chlorophyll derivatives were established for Brazilian EVOOs for the first time in this study. Brazilian cv. Koroneiki EVOOs from the Southeast showed the highest concentrations of total secoiridoids among the 15 analyzed samples. Arbequina oils from Brazil were the richest in terms of lignans. These compositional features highlight the potential bioactivity of selected Brazilian EVOOs, although this deserves confirmation in future studies by considering further commercial samples and crop seasons. In general, simple phenols and related analytes represented approx. 3.6 to 20% of the total phenolic compounds in every sample. Among the samples from Brazil, most EVOOs from the Southeast presented higher contents of pentacyclic triterpenes when compared to samples from the South. Regarding pigments, pheophytin *a* and β-carotene were found at higher concentrations. Overall, the pheophytin *a* content in Brazilian EVOOs accounted for 53–92% of the total chlorophyll derivatives, and its contents in cv. Koroneiki samples were 4-fold and 2-fold higher than those of cv. Arbequina from Brazil and Spain, respectively.

Future studies regarding commercial Brazilian EVOOs’ composition would further contribute to robustly determining the chemical profile patterns that could be used as biomarkers of origin and quality.

## Figures and Tables

**Table 1:** Quality parameters, *p*-anisidine value, antioxidant capacity, oxidative stability index, and total phenolic content of the studied extra virgin olive oils (EVOOs).

EVOO Samples	Free Acidity (% 18:1)	Peroxide Value (mEq O_2_/kg)	k_232_	k_270_	*p*-Anisidine Value	Antioxidant Capacity (mmol TE/kg)	Oxidative Stability Index (h)	Total Phenolic Content (mg GAE/100 g)
**Brazilian cv. Arbequina**								
Southeast A	0.18 ± 0.00 ^a,g,k^	8.62 ± 0.07 ^a^	2.31 ± 0.01 ^a,i^	0.22 ± 0.01 ^a^	4.36 ± 0.12 ^a^	2.52 ± 0.02 ^a^	12.75 ± 0.12 ^a^	4.42 ± 0.39 ^a^
Southeast B	0.36 ± 0.03 ^b^	19.10 ± 0.27 ^b^	2.88 ± 0.06 ^b^	0.18 ± 0.01 ^b,c^	4.08 ± 0.02 ^a^	2.97 ± 0.04 ^b^	13.08 ± 0.19 ^a^	6.53 ± 0.11 ^b,f,i^
South C	0.04 ± 0.00 ^c^	7.27 ± 0.01 ^c,g^	1.98 ± 0.01 ^c^	0.14 ± 0.01 ^d,f,g,h,i^	5.78 ± 0.06 ^b^	2.35 ± 0.04 ^a^	15.58 ± 0.17 ^b^	4.31 ± 0.22 ^a^
South D	0.20 ± 0.02 ^a,i,l^	9.48 ± 0.12 ^d^	2.29 ± 0.01 ^a^	0.13 ± 0.00 ^d,e,j,k,l^	5.28 ± 0.05 ^c^	3.08 ± 0.07 ^a^	13.21 ± 0.07 ^a^	5.52 ± 0.47 ^a,i^
South E	0.11 ± 0.00 ^d^	9.70 ± 0.08 ^d^	2.36 ± 0.01 ^d,i^	0.15 ± 0.01 ^e,m,n^	7.16 ± 0.07 ^d^	2.40 ± 0.09 ^a^	16.93 ± 0.38 ^c^	6.04 ± 0.52 ^b,i^
**Brazilian cv. Koroneiki**								
Southeast B	0.39 ± 0.01 ^b^	3.89 ± 0.01 ^e^	1.65 ± 0.05 ^e^	0.13 ± 0.00 ^f,j,o,p^	2.80 ± 0.06 ^e^	3.29 ± 0.09 ^b^	64.72 ± 0.30 ^d^	18.9 ± 0.78 ^d^
Southeast F	0.44 ± 0.02 ^e^	6.95 ± 0.04 ^c^	1.47 ± 0.01 ^f^	0.16 ± 0.00 ^m,q^	1.59 ± 0.02 ^f^	3.19 ± 0.09 ^b^	43.60 ± 0.13 ^e^	11.3 ± 0.01 ^e^
South C	0.15 ± 0.01 ^d,g^	4.06 ± 0.10 ^e^	1.50 ± 0.01 ^f^	0.12 ± 0.00 ^f,j,r^	7.13 ± 0.14 ^d^	2.36 ± 0.11 ^a^	41.09 ± 0.73 ^f^	7.38 ± 0.32 ^b,f,g^
South G	0.18 ± 0.00 ^f,g,l^	9.22 ± 0.01 ^a,d^	2.09 ± 0.01 ^g^	0.19 ± 0.00 ^b^	10.8 ± 0.16 ^g^	2.38 ± 0.05 ^a^	32.75 ± 0.42 ^g^	9.33 ± 0.24 ^c,h^
South H	0.16 ± 0.01 ^g^	7.63 ± 0.09 ^c,g^	2.07 ± 0.02 ^g,j^	0.15 ± 0.00 ^g,m,q,s^	8.07 ± 0.31 ^h^	2.27 ± 0.06 ^a^	23.67 ± 0.50 ^h^	6.08 ± 0.29 ^b,i^
**Spanish cv. Arbequina**								
Catalonia I	0.28 ± 0.02 ^h^	13.90 ± 0.47 ^f^	2.87 ± 0.00 ^b^	0.14 ± 0.00 ^h,k,n,o,s^	4.42 ± 0.09 ^a^	3.28 ± 0.14 ^b^	12.52 ± 0.01 ^a^	7.81 ± 0.35 ^f,g^
Catalonia J	0.23 ± 0.03 ^i^	7.68 ± 0.44 ^g^	2.20 ± 0.01 ^h^	0.17 ± 0.00 ^c^	7.44 ± 0.05 ^d^	3.08 ± 0.21 ^b^	26.45 ± 0.35 ^i^	11.8 ± 0.05 ^e^
Catalonia K	0.31 ± 0.00 ^h^	5.83 ± 0.48 ^h^	2.00 ± 0.00 ^c,j^	0.14 ± 0.00 ^h,k,n,o,s^	4.90 ± 0.06 ^c^	3.04 ± 0.10 ^b^	16.58 ± 0.33 ^c,b^	8.06 ± 0.10 ^g,h^
Catalonia L	0.58 ± 0.00 ^j^	9.52 ± 0.05 ^d^	2.18 ± 0.01 ^h^	0.15 ± 0.00 ^h,n,q,s^	6.68 ± 0.23 ^i^	2.99 ± 0.27 ^b^	16.81 ± 0.23 ^c^	9.63 ± 0.08 ^c^
Catalonia M	0.22 ± 0.01 ^f,i,k^	9.52 ± 0.07 ^d^	2.18 ± 0.02 ^h^	0.13 ± 0.00 ^i,k,l,p,r^	5.18 ± 0.16 ^c^	3.07 ± 0.06 ^b^	20.52 ± 0.39 ^j^	8.47 ± 0.31 ^c,g^
**Established limits ^1^**	0.80	20.0	2.50	0.22	10.0 *	-	-	-

Results expressed as the mean value ± standard deviation of triplicates. ^1^ Established limits [28]. * Value recommended in the literature for vegetable oils [30]. Superscripted letters in columns indicate significant differences between samples (*p* < 0.05; one-way ANOVA followed by Tukey’s post-hoc test).

**Table 2:** Fatty acid composition (g/100 g) of Brazilian cv. Arbequina and cv. Koroneiki and Spanish cv. Arbequina EVOOs determined by gas chromatography (GC)-flame ionization detector (FID).

EVOO Samples	Fatty Acid Composition (g/100 g)						
	16:0	16:1*n-*7	18:0	18:1*n-*9	18:2*n-*6	18:3*n-*3	M: P_ratio_
**Brazilian cv. Arbequina**							
Southeast A	14.3 ± 0.30 ^a,d,f^	1.59 ± 0.05 ^a^	1.51 ± 0.06 ^a,b^	73.1 ± 0.15 ^a^	8.91 ± 0.16 ^a^	0.57 ± 0.04 ^a,c,d^	7.89 ± 0.16 ^a^
Southeast B	15.1 ± 0.25 ^a,b,g^	0.11 ± 0.02 ^b^	1.18 ± 0.41 ^a^	73.2 ± 0.14 ^a^	9.85 ± 0.03 ^b^	0.61 ± 0.00 ^a,c,e^	7.00 ± 0.02 ^b^
South C	16.7 ± 0.39 ^a,h^	2.05 ± 0.09 ^c^	1.40 ± 0.04 ^a,b^	70.0 ± 0.50 ^b^	9.32 ± 0.04 ^c^	0.57 ± 0.06 ^a,c,d^	7.28 ± 0.05 ^b, j^
South D	16.9 ± 1.66 ^b,h,i^	0.10 ± 0.00 ^b^	1.39 ± 0.58 ^a,b^	66.6 ± 1.10 ^c^	14.3 ± 0.20 ^d^	0.75 ± 0.01 ^b^	4.44 ± 0.01 ^c^
South E	17.1 ± 0.39 ^b,h,i^	1.92 ± 0.04 ^d^	1.60 ± 0.03 ^a,b^	70.1 ± 0.35 ^b^	8.68 ± 0.09 ^a^	0.61 ± 0.04 ^a,c,e,f^	7.75 ± 0.06 ^a, j^
**Brazilian cv. Koroneiki**							
Southeast B	11.0 ± 0.49 ^c,e^	0.04 ± 0.00 ^b^	1.10 ± 0.23 ^a,b^	83.8 ± 0.36 ^d^	3.52 ± 0.02 ^e^	0.62 ± 0.00 ^a,c,e,g^	20.3 ± 0.15 ^d^
Southeast F	8.54 ± 1.21 ^c^	0.04 ± 0.00 ^b^	1.23 ± 0.65 ^a,b^	86.0 ± 0.72 ^e^	3.55 ± 0.05 ^e^	0.64 ± 0.00 ^a,e,h^	20.6 ± 0.37 ^d^
South C	13.1 ± 0.26 ^d,e,g,j^	0.81 ± 0.06 ^e^	1.28 ± 0.13 ^a,b^	78.8 ± 0.34 ^f^	4.66 ± 0.12 ^f^	0.68 ± 0.05 ^b,f,g,h,i^	14.9 ± 0.50 ^e^
South G	14.1 ± 0.37 ^a,j,k^	0.86 ± 0.09 ^e,f^	2.10 ± 0.09 ^a^	76.7 ± 0.42 ^g,h,i^	5.69 ± 0.12 ^g^	0.58 ± 0.04 ^a,c,d,e^	12.3 ± 0.20 ^f^
South H	14.5 ± 0.19 ^a,i,j,k^	1.04 ± 0.01 ^f^	1.54 ± 0.16 ^a^	75.2 ± 0.24 ^g,j^	6.27 ± 0.09 ^h^	0.92 ± 0.06 ^j^	10.6 ± 0.25 ^g^
**Spanish cv. Arbequina**							
Catalonia I	15.3 ± 0.21 ^a,i,j^	0.21 ± 0.17 ^b^	1.85 ± 0.49 ^a,b^	69.8 ± 0.48 ^b^	12.3 ± 0.15 ^i^	0.55 ± 0.01 ^a,c,d^	5.44 ± 0.06 ^h^
Catalonia J	11.4 ± 0.76 ^e^	0.19 ± 0.11 ^b^	2.27 ± 0.94 ^a,b^	77.6 ± 0.59 ^f,h^	8.07 ± 0.10 ^j^	0.53 ± 0.01 ^c,d^	9.04 ± 0.14 ^i^
Catalonia K	15.5 ± 1.47 ^a,i,j^	0.13 ± 0.00 ^b^	1.61 ± 0.50 ^a,b^	68.5 ± 0.88 ^b^	13.6 ± 0.08 ^k^	0.66 ± 0.01 ^b,e^	4.80 ± 0.03 ^c^
Catalonia L	12.3 ± 1.59 ^e,f,k^	0.12 ± 0.01 ^b^	2.63 ± 0.66 ^b^	74.0 ± 1.17 ^a,j^	10.4 ± 0.26 ^l^	0.61 ± 0.01 ^a,c,e,i^	6.72 ± 0.08 ^b^
Catalonia M	11.5 ± 0.76 ^e^	0.11 ± 0.01 ^b^	1.35 ± 0.65 ^a,b^	77.6 ± 0.84 ^f,i^	8.97 ± 0.10 ^a,c^	0.50 ± 0.00 ^d^	8.21 ± 0.06 ^a^

Results were expressed as the mean ± standard deviation (*n* = 3). M:P_ratio_, monounsaturated fatty acid to polyunsaturated fatty acid ratio. Superscript letters in columns indicate significant differences between samples (*p* < 0.05; one-way ANOVA followed by Tukey’s post-hoc test).

**Table 3:** Geographical origin and commercial brands of the studied EVOO samples.

Country of Origin	Cultivar	Country Region	Designation	Latitude	Longitude	Altitude (m)
		Southeast	A	22°41′18″ S	45°44′11″ W	874
		Southeast	B	21°57′35″ S	44°53′29″ W	893
	Arbequina	South	C	31°23′44″ S	52°41′11″ W	408
		South	D	31°28′36″ S	53°40′45″ W	197
Brazil		South	E	30°53′23″ S	55°31′56″ W	215
		Southeast	B	21°57′35″ S	44°53′29″ W	893
		Southeast	F	21°55′46″ S	44°36′07″ W	1155
	Koroneiki	South	C	31°23′44″ S	52°41′11″ W	408
		South	G	30°30′59″ S	53°29′12″ W	430
		South	H	30°42′16″ S	52°06′36″ W	134
		Catalonia	I	41°36′51″ N	00°37′33″ W	169
		Catalonia	J	41°38′49″ N	01°08′21″ W	385
Spain	Arbequina	Catalonia	K	41°32′25″ N	00°55′07″ W	304
		Catalonia	L	41°32′25″ N	00°55′07″ W	304
		Catalonia	M	41°38′49″ N	01°08′21″ W	385

Latitude, longitude, and altitude of Brazilian samples were obtained from the website https://www.cidade-brasil.com.br/, and for Spanish samples, the website https://es.db-city.com/was used as a source.

## Supplementary Materials

The following are available online: Figure S1a: Representative extracted ion chromatograms (EIC) of the studied EVOOs determined by RP-LC-MS; Figure S1b: Representative chromatograms of the studied EVOOs determined by NP-LC-DAD/FLD; Table S1: Summary of data and references reporting the chemical profile and quality indices of Brazilian olive oils (OO), virgin olive oils (VOO), and extra virgin olive oils (EVOO); Table S2a: EVOO minor components determined by RP-LC-MS (mg/kg); Table S2b: EVOO minor components determined by NP-LC-DAD/FLD (mg/kg); Table S3: Volatile and semi-volatile compounds (µg/g) of the studied EVOOs determined by SPME-GC-MS; Table S4a: Identification parameters of minor components determined by RP-LC-MS in the studied samples; Table S4b: Identification parameters of minor components determined by NP-LC-DAD/FLD in the studied samples; Table S5a: Analytical parameters of the RP-LC-MS method; Table S5b: Analytical parameters of the NP-LC-DAD/FLD method; Table S6: Volatile compounds detected in the studied EVOOs determined by SPME-GC-MS, including identification parameters and related aroma notes.
