# Supplementary material for: Evaluating Quality Parameters, the Metabolic Profile, and Other Typical Features of Selected Commercial Extra Virgin Olive Oils from Brazil
Source: Molecules. 2020 Sep 13;25(18):4193. doi: 10.3390/molecules25184193 (PMC7570599; doi:10.3390/molecules25184193)
Supplement: Supplementary file 1 [file molecules-25-04193-s001.pdf]

Supplementary Materials

Evaluating Quality Parameters, the Metabolic Profile, and Other Typical Features of Selected Commercial Extra Virgin Olive Oils from Brazil

Aline Gabrielle Alves de Carvalho [1], Lucía Olmo-García [2], Bruna Rachel Antunes Gaspar [1], Alegría Carrasco-Pancorbo [2,*,†], Vanessa Naciuk Castelo-Branco [3,†] and Alexandre Guedes Torres [1,†]

[1] Laboratory of Nutritional Biochemistry and Food Science, Lipid Biochemistry and Lipidomics Laboratory, Institute of Chemistry, Federal University of Rio de Janeiro, Rio de Janeiro 21941-902, Brazil; alinegac@gmail.com (A.G.A.d.C.); brunargaspar@gmail.com (B.R.A.G.); torres@iq.ufrj.br (A.G.T.)

[2] Department of Analytical Chemistry, Faculty of Sciences, University of Granada, Ave. Fuentenueva s/n, E-18071 Granada, Spain; luciaolmo@ugr.es (L.O.-G.); alegriac@ugr.es (A.C.-P.)

[3] Laboratory of Food Biotechnology, Fluminense Federal University, Niteroi 24230-340, RJ, Brazil; vanessanaciuk@id.uff.br (V.N.C.-B)

* Correspondence: alegriac@ugr.es; Tel.: +34-958-242-785

† These authors contributed equally to this work.

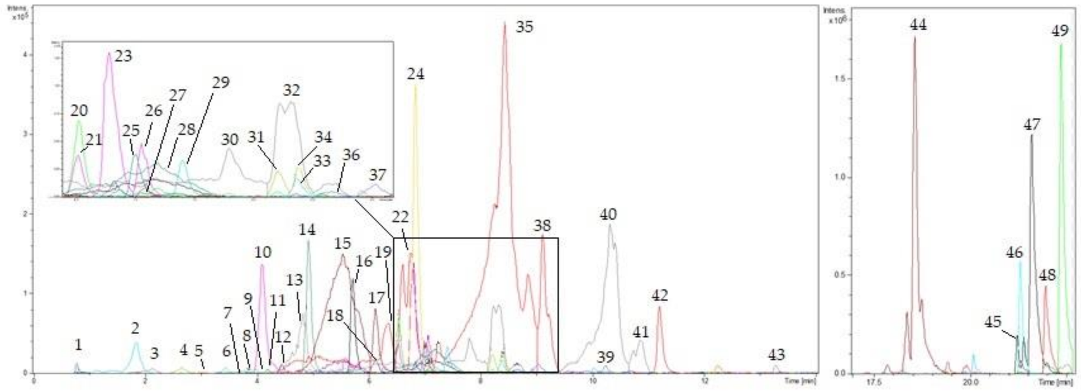

Figure S1a. Representative extracted ion chromatograms (EIC) of the studied EVOOs determined by RP-LC-MS. 1. Quinic acid; 2. Hydroxytyrosol; 3. Eudesmic acid; 4. Tyrosol; 5. Vanillic acid; 6. Decarboxymethyl elenolic acid; 7. *p*-Coumaric acid; 8. Vanillin; 9. Ferulic acid; 10. Desoxy elenolic acid; 11. Hydroxy elenolic acid; 12. Hydroxytyrosol acetate; 13. Elenolic acid; 14. Hydroxy decarboxymethyl oleuropein aglycone; 15. Decarboxymethyl oleuropein aglycone (isomer 1); 16. Luteolin; 17. Decarboxymethyl oleuropein aglycone (isomer 2); 18. Syringaresinol; 19. Oleuropein aglycone (isomer 1); 20. Pinoresinol; 21. 10-Hydroxy oleuropein aglycone 1; 22. Oleuropein aglycone (isomer 2); 23. 10-Hydroxy oleuropein aglycone (isomer 2); 24. Acetoxypinoresinol; 25. Apigenin; 26. 10-Hydroxyoleuropein aglycone (isomer 3); 27. Naringenin; 28. Decarboxymethyl ligstroside aglycone; 29. Diosmetin; 30. Ligstroside aglycone (isomer 1); 31. Methyl decarboxymethyl oleuropein aglycone (isomer 1); 32. Ligstroside aglycone (isomer 2); 33. Dehydro oleuropein aglycone; 34. Methyl decarboxymethyl oleuropein aglycone (isomer 2); 35. Oleuropein aglycone (isomer 3); 36. Methyl oleuropein aglycone (isomer 1); 37. Methyl oleuropein aglycone (isomer 2); 38. Oleuropein aglycone (isomer 4); 39. Dehydro ligstroside aglycone. 40. Ligstroside aglycone (isomer 3); 41. Ligstroside aglycone (isomer 4); 42. Oleuropein aglycone (isomer 5); 43. Ligstroside aglycone (isomer 5). 44. Maslinic acid; 45. Betulinic acid; 46. Linolenic acid; 47. Oleanolic acid; 48. Palmitoleic acid; 49. Linoleic acid.

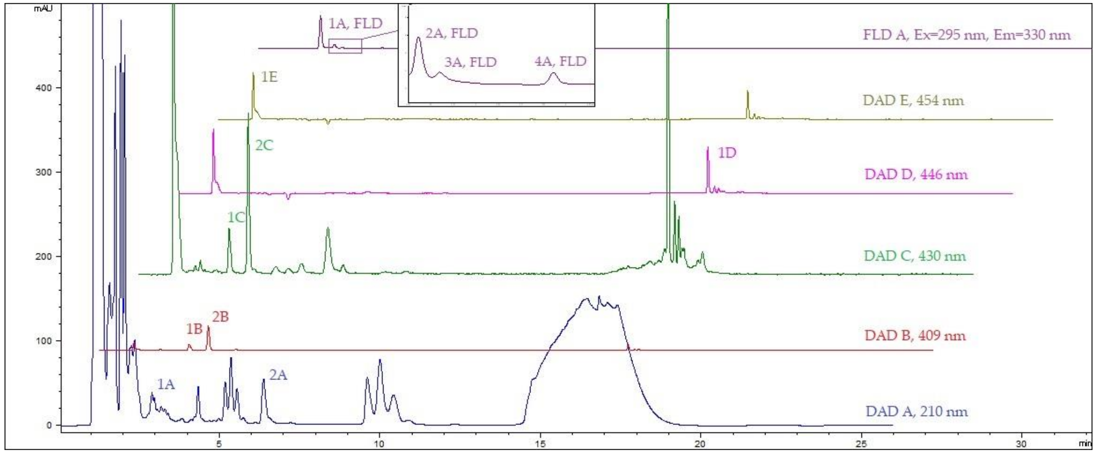

Figure S1b. Representative chromatograms of the studied EVOOs determined by NP-LC-DAD/FLD. DAD A- Phytosterols: 1A. Lupeol; 2A. β-Sitosterol + free sterols. Peak around 14-20 min does not interfere in phytosterols identification/quantification in any sample. **DAD B** - Chlorophylls *a* derivatives: 1B. Pyropheophytin *a*; 2B. Pheophytin *a*. **DAD C** - Chlorophylls *b* derivatives: 1C. Pyropheophytin *b*; 2C. Pheophytin *b*. **DAD D** – Carotenoid: 1D. Lutein. **DAD E** – Carotenoid: 1E. β-Carotene. **FLD A** - Tocopherols: 1A, FLD. α-Tocopherol; 2A, FLD. β-Tocopherol; 3A, FLD. γ-Tocopherol; 4A, FLD. δ-Tocopherol.

Table S1. Summary of data and references reporting the chemical profile and quality indices of Brazilian olive oils (OO), virgin olive oils (VOO) and extra virgin olive oils (EVOO).

Types of olive oil♦	Olive varieties	Production regions	Harvest year(s)	Number of samples	Components analyzed and quality indices[1]	Reference♣
VOO, experimental	2010 *harvest, monovarietals*: MGS Grappolo 561, Cornicabra, Tafahi 390, Grappolo 575, Arbequina, Alto D'Ouro, Negroa, MGS Neblina and JB1<br>2011 *harvest, monovarietals*: MGS Mariense (Maria da Fé), Mission, Grappolo 575, Arbequina, Alto D'Ouro, Negroa, MGS Neblina and JB1	*Southeast of Brazil*: Mantiqueira mountain range (Minas Gerais)	2010<br>2011	17	Fatty acids (8)<br>Phenolic compounds (4)<br>Tocopherols (3)	[Ballus et al, 2014][7]
VOO, experimental	*Monovarietals*: Arbequina, Koroneiki, Arbosana, Grappolo, Manzanilla, Coratina, Frantoio and MGS Mariense	*Southeast of Brazil*: Minas Gerais<br>*South of Brazil*: Rio Grande do Sul and Santa Catarina	2011<br>2012	25	Phenolic compounds (19)	[Ballus et al, 2015][15]
EVOO, experimental	*Monovarietal*: Arbequina	*Brazil*: *Southeast* (Minas Gerais) and *South* (Rio Grande do Sul). *Spain*: Granada, Jaén, Málaga, Cádiz, Sevilla, Albacete, Toledo, Valladolid and Lérida.	2014/15	Brazil, 2<br>Spain, 9	Fatty acids (12)<br>Total pigments (2)<br>Quality indices<br>Oxidative stability	[Borges et al, 2017a][16]
EVOO, experimental	*Monovarietal*: Arbequina	*Brazil*: *Southeast* (Minas Gerais) and *South* (Rio Grande do Sul). *Spain*: Granada, Jaén, Málaga, Cádiz, Sevilla, Albacete, Toledo, Valladolid and Lérida.	2014/15	Brazil, 2<br>Spain, 9	Coenzyme Q10<br>Tocopherols (3)<br>Phenolic compounds (6)	[Borges et al, 2017a][17]
OO, experimental	*Monovarietals*: Arbequina, Coratina, Frantoio and Koroneiki	*South of Brazil*: Rio Grande do Sul	2013/14	4	Tocopherols (3)<br>Total pigments (2)<br>Fatty acids (9)<br>Total phenolic compounds<br>Quality indices	[Bruscatto et al, 2017][18]

Types of olive oil♦	Olive varieties	Production regions	Harvest year(s)	Number of samples	Components analysed and quality indices[1]	Reference
VOO, commercial	*Monovarietals*: Arbequina, Grappolo, Koroneiki, and Coratina<br>*Blends*: Arbequina and Arbosana, Arbequina and Grappolo	*Southeast of Brazil*: Minas Gerais and São Paulo<br>*South of Brazil*: Paraná	2016/17	12	Fatty acids (12)<br>Fatty acid alkyl esters<br>Total pigments (2)<br>Tocopherols (total)<br>Phenolic compounds (13)<br>Volatile compounds (17)<br>Quality indices<br>Antioxidant activity<br>Sensory panel test	[Zago et al, 2019][19]
VOO, experimental	*Monovarietals*: Frantoio, Arbequina, Mission, Arbosana, Maria da Fé, Grappolo 541, and Ascolano 315<br>*Blend*: Grappolo 541 and Arbequina	*Southeast of Brazil*: Mantiqueira mountain range	Not specified	8	Fatty acids (12)<br>Volatile compounds (38)<br>Quality indices<br>Sensory test	[Rodrigues et al, 2019][20]
EVOO, experimental	*Monovarietals*: Arbequina, Arbosana, Coratina, Frantoio, Koroneiki, and Picual	*South of Brazil*: Rio Grande do Sul	2017<br>2018	6	Fatty acids (11)<br>Total pigments (2)<br>Tocopherols (3)<br>Phenolic compounds (19)<br>Quality indices	[Crizel et al, 2020][21]
EVOO, experimental	*Monovarietals*: Arbequina, Empeltre, Coratina, Grappolo, Koroneiki, and Maria da Fé	*Southeast of Brazil*: Minas Gerais	2015	24	Fatty acids (4)<br>Chemical stability during storage, by UV-Vis and NIR spectroscopy<br>Quality indices*	[Gonçalves et al, 2020][22]

[1]Quality indices: Free acidity, peroxide value, specific extinction coefficients. *Also included iodine value analysis. ♦According to the nomenclature used by the authors of the cited contribution. When stated "experimental oils" indicates that the samples were obtained by using an Abencor or similar system. ♣ We have added the references

both with a number (as in the text of the manuscript) as well as with the name of the first author and year of publication, for ease of reading and finding information. References: Ballus, C.A. et al. *Food Res. Int.* **2014,** *62*, 74-83; Ballus, C.A. et al. *Food chem.* **2015**, *170*, 366-377; Borges, T.H. et al. *Food chem.* **2017a**, *215*, 454-462. Borges, T.H. et al. *J. Food Compost. Anal.* **2017b,** *63*, 47-54; Bruscatto, M.H. et al. *Pesq. Agropec. Bras.* **2017**, *52(12)*, 1231-1240; Zago, L. et al. *Food Res. Int.* **2019**, *126*, 108588; Rodrigues, J.F. et al. *Bragantia* **2019,** *78(4)*, 479-489; Crizel, R.L. et al. *Eur J Lipid Sci Tech.* **2020**, *122(4)*, 1900347; Gonçalves, T.R. et al. *Food Anal. Methods* **2020**, *13(1)*, 86-96.

Table S2a. EVOOs minor components determined by RP-LC -MS (mg/kg).

	**Brazilian cv. Arbequina**					**Brazilian cv. Koroneiki**					**Spanish cv. Arbequina**				
	**Southeast**		**South**			**Southeast**		**South**			**Catalonia**				
	**A**	**B**	**C**	**D**	**E**	**B**	**F**	**C**	**G**	**H**	**I**	**J**	**K**	**L**	**M**
**Simple phenols, phenolic acids and related substances**															
Quinic acid	n.q.	0.13*	n.d.	0.52	n.q.	0.29	0.16	0.04*	0.13*	0.18*	n.d.	n.d.	n.d.	n.d.	n.d.
Oxidized Hydroxytyrosol	n.d.	n.d.	n.d.	n.d.	n.d.	n.d.	n.d.	n.d.	n.d.	n.d.	n.d.	n.d.	n.d.	n.d.	0.07
Hydroxytyrosol	1.47*	3.57	n.q.	1.80	0.61	4.35	9.12	7.49	9.20	0.88*	2.20*	3.99	1.37	3.37	1.42
Eudesmic acid	0.94*	7.01	1.09*	7.34	1.70	8.59	8.12	1.46	1.52	n.d.	6.59	7.83	7.32*	7.07	6.85
Tyrosol	0.90*	1.38	0.72*	0.95	0.78*	1.38	2.88	2.68	2.74	2.75	2.71	2.43	1.38	2.78	0.98
Vanillic acid	0.77*	1.38	1.70*	0.38*	1.33*	0.66	0.53	1.14	0.66*	0.79	0.20	0.25*	0.35	0.16*	0.27*
p-coumaric acid	n.q.	0.49	0.10	n.d.	n.d.	n.d.	0.22	n.d.	n.d.	n.d.	n.d.	0.21*	0.25*	n.d.	n.d.
Vanillin	0.24	0.12	n.q.	0.03*	n.q.	0.06*	0.06*	n.d.	n.d.	n.d.	0.02	0.01	n.q.	0.19*	0.08*
Ferulic acid	n.d.	n.d.	n.d.	n.d.	n.d.	0.44*	0.39*	n.d.	n.d.	n.d.	n.d.	n.d.	n.d.	n.d.	n.d.
Hydroxytyrosol acetate	1.41	9.38	0.62*	4.32*	0.50	1.24*	0.50*	n.d.	1.19	0.52	1.29	2.38	1.61*	0.39	1.47*
**Total simple phenols, phenolic acids and related substances**	5.73	23.5	4.23	15.3	4.92	17.0	22.0	12.8	15.4	5.12	13.0	17.1	12.3	14.0	11.1
**Elenolic acid-related substances**															
Decarboxymethyl elenolic acid	0.09	0.31	0.09*	n.d.	0.04*	11.2	0.24	0.13	n.d.	n.d.	n.d.	0.22	n.d.	0.13	n.d.
Desoxy elenolic acid	0.76*	0.68	n.d.	0.94	0.98	5.54*	3.88	2.78	3.25	1.88	0.20	0.77	0.20*	0.17*	0.14
Hydroxy elenolic acid	0.23*	0.10	0.18*	0.09	0.46	0.14	0.11	0.29	0.64	0.35	0.13	0.30	0.08	0.19	0.32
Elenolic acid	1.65*	0.75	3.36*	1.10	7.15*	4.30	4.21	4.05	7.92	3.05	0.86	2.40	0.78	1.76	1.64*
**Total elenolic acid-related substances**	2.73	1.84	3.63	2.13	8.63	21.2	8.44	7.25	11.8	5.28	1.19	3.69	1.06	2.25	2.10

	Brazilian cv. Arbequina					Brazilian cv. Koroneiki					Spanish cv. Arbequina				
	Southeast		South			Southeast		South			Catalonia				
	A	B	C	D	E	B	F	C	G	H	I	J	K	L	M
**Oleuropein aglycone-related substances**															
Hydroxy decarboxymethyl oleuropein aglycone	1.13*	7.22	0.59*	3.33	3.38	5.16	1.58	8.54	8.56	1.99	3.21	8.38	1.67	2.44	7.61
Decarboxymethyl oleuropein aglycone isomer 1	1.47	7.88	2.12*	6.63	3.42*	50.9	12.1	16.9	15.1	1.66	7.26	20.2	14.5*	8.25	12.5
Decarboxymethyl oleuropein aglycone isomer 2	0.50*	1.42	0.50*	1.53	1.30	1.59	0.58	1.90	2.55	1.52	2.95	4.22*	1.37	2.13	3.97
Oleuropein aglycone isomer 1	0.06*	0.36*	0.16*	0.36*	0.68	20.5*	14.9*	4.99	7.18	0.75*	0.32*	0.16*	1.01*	1.40	0.18*
Oleuropein aglycone isomer 2	0.19*	0.78	0.38*	0.74*	0.90	23.0	14.3*	1.23*	1.14	0.52*	0.11*	0.31*	0.40*	2.15*	n.d.
Oleuropein aglycone isomer 3	3.91	7.54*	4.94*	6.63*	7.21*	249	120*	77.5	84.4	17.4	8.92	31.0	23.3*	44.5	8.83
Oleuropein aglycone isomer 4	0.14*	0.42*	0.39*	0.46	0.38*	7.55	4.73	2.64	2.97	1.05	0.43*	1.70	1.31*	2.76*	0.46*
Oleuropein aglycone isomer 5	0.09*	0.33	0.41*	0.49	0.40*	2.18	2.10	0.99	0.62	0.36	0.33	0.50	0.32*	0.35	0.27
10-Hydroxyoleuropein aglycone isomer 1	0.31	1.12	0.14*	0.40*	0.37	1.13	0.58*	0.67	1.37	0.60	0.73	1.15	0.30	0.61*	0.90
10-Hydroxyoleuropein aglycone isomer 2	0.41	0.60	0.13*	0.37*	0.51	4.46	2.74	2.53	7.65	2.42	1.49	4.63	0.86	2.68	2.07
10-Hydroxyoleuropein aglycone isomer 3	0.65*	1.01	0.41*	0.76*	0.94	0.56	0.69*	1.00	1.98	1.15	0.62	1.24	0.16	0.41*	0.76
Methyl decarboxymethyl oleuropein aglycone 1	0.04	0.07*	0.01*	0.11	0.03	0.63	0.36	0.20	0.57	0.24	0.34	0.66	0.18	0.61	0.40
Methyl decarboxymethyl oleuropein aglycone 2	0.05	0.09	0.03*	0.13	0.07*	0.68	0.40	0.27	0.51	0.20	0.17	0.30	0.09	0.27	0.15*
Dehydro oleuropein aglycone	n.d.	n.d.	0.08*	0.08	0.15	0.24*	1.17*	0.16	0.29	0.16*	0.42*	0.53	0.37	1.06	0.60

	Brazilian cv. Arbequina					Brazilian cv. Koroneiki					Spanish cv. Arbequina				
	Southeast		South			Southeast		South			Catalonia				
	A	B	C	D	E	B	F	C	G	H	I	J	K	L	M
**Oleuropein aglycone-related substances (cont.)**															
Methyl oleuropein aglycone isomer 1	0.27*	0.16*	0.15*	0.25*	0.46*	0.21*	0.13*	0.11	0.12	0.30*	0.12*	0.27*	0.12*	n.d.	0.08*
Methyl oleuropein aglycone isomer 2	0.58	0.70*	0.46*	1.32	1.33	0.33	0.20*	0.65	0.29	0.36*	0.58	0.84	0.73*	n.d.	0.32
**Total oleuropein aglycone-related substances**	9.80	29.7	10.9	23.6	21.5	368	177	120	135	30.7	28.0	76.1	46.7	69.6	39.1
**Ligstroside aglycone-derivatives**															
Decarboxymethyl ligstroside aglycone	0.41	0.57*	0.92*	1.61	0.97*	4.13	1.47	3.98	3.12	0.67*	2.43	3.79	3.44	3.01	3.08
Ligstroside aglycone isomer 1	0.20*	0.12*	0.21*	0.19*	0.30*	6.89	3.79*	0.35*	0.48*	0.99*	0.11	0.68*	0.15	0.48*	0.09
Ligstroside aglycone isomer 2	0.29*	0.47*	0.40*	0.53*	0.49*	3.50	2.52	1.48*	2.52	0.35	0.40	1.44*	0.28*	1.41	0.46*
Ligstroside aglycone isomer 3	1.03	0.63*	0.80	1.85*	0.98	31.7	10.1	5.86	6.45	7.87	3.63	9.33*	8.49*	26.0	4.78*
Ligstroside aglycone isomer 4	0.08*	0.11*	0.05*	0.07*	0.05*	3.02	1.53	0.80	0.88	0.62*	0.14*	0.99*	0.76*	1.87	0.27*
Ligstroside aglycone isomer 5	n.d.	n.d.	n.d.	0.09	n.d.	0.28	0.16	n.d.	n.d.	n.d.	0.08*	0.12	0.08	0.08	0.07
Dehydro ligstroside aglycone	n.q.	n.d.	0.01*	0.06*	0.02*	n.d.	0.45*	0.04	0.09	0.12	0.17	0.20	0.16	0.36	0.24
**Total ligstroside aglycone-derivatives**	2.01	1.90	2.39	4.40	2.81	49.5	20.0	12.5	13.5	10.6	6.96	16.6	13.4	33.2	8.99
**Flavonoids**															
Luteolin	1.18	1.97	2.42*	3.00	2.88	1.55	2.16	2.78	2.87	1.42	1.35	1.77	1.98	1.41	2.32
Apigenin	n.d.	0.33*	0.88	0.38	0.84	0.44	0.92*	1.06	0.85	0.28	0.41*	0.39	0.59*	0.37	0.59

	Brazilian cv. Arbequina					Brazilian cv. Koroneiki					Spanish cv. Arbequina				
	Southeast		South			Southeast		South			Catalonia				
	A	B	C	D	E	B	F	C	G	H	I	J	K	L	M
**Flavonoids (cont.)**															
Naringenin	n.d.	0.05	0.07*	0.11*	0.12*	0.10	0.14*	n.q.	n.q.	n.q.	0.10	0.11	0.12	0.04*	0.25*
Diosmetin	0.75*	0.85*	1.00	1.36*	1.55	0.30	0.63*	0.65	0.65	0.62	1.14*	0.90*	1.21	0.42*	1.23
**Total flavonoids**	1.93	3.20	4.37	4.85	5.39	2.39	3.85	4.49	4.37	2.32	3.00	3.17	3.90	2.24	4.39
**Lignans**															
Syringaresinol	1.69*	2.13	n.d.	1.85	1.50	0.19*	0.44	0.96*	0.81	0.68	1.06	0.81	2.18	1.09	0.79
Pinoresinol	6.64	4.38	4.02*	4.53	4.47	1.79	3.06	1.98	2.54	2.89	2.75	2.57	2.94	2.87	2.68
Acetoxypinoresinol	43.5	49.5	36.2*	42.8	41.6	15.3*	15.9	18.4	23.5	29.5	23.0	14.9	25.5	5.28	18.5
**Total lignans**	51.8	56.0	40.2	49.2	47.6	17.3	19.4	21.3	26.9	33.1	26.8	18.3	30.6	9.24	22.0
**Triterpenic compounds**															
Maslinic acid	34.9*	92.3	21.3	74.0	32.2	81.3	88.2	17.5	44.0	18.6	39.7	41.7	75.5	57.5	28.6
Betulinic acid	13.1	15.2	6.96	15.2*	14.9	16.1	15.1	3.94*	7.22	3.83	6.16*	7.23*	17.7	10.6*	5.24*
Oleanolic acid	4.93	12.6	3.38	5.65*	4.64	10.8	10.5	2.03*	3.44	2.25	3.73*	4.68	11.9	4.70*	3.84*
**Total triterpenic compounds**	52.9	120	31.6	94.9	51.7	108	114	23.5	54.7	24.7	49.6	53.6	105	72.8	37.7
**Free fatty acids**															
Linolenic acid	2.27*	0.69	0.35*	0.76	0.31	0.43	1.05	0.63	1.36	0.99	1.09*	1.11	0.93*	3.10*	1.25
Palmitoleic acid	47.6	12.9	5.68*	16.0*	4.88	2.67	9.61	3.73	13.3	9.20	15.6*	12.2	10.6	23.3*	9.42*
Linoleic acid	264*	86.3	23.9	96.3*	23.9	34.5	62.0	22.4	121	37.7	129	104	82.1*	188*	81.3*
**Total free fatty acids**	314	99.9	29.9	113	29.1	37.6	72.7	26.8	136	47.9	146	117	93.6	214	92.0

Results expressed as mean value of two independent replicates. n.d. – not detected. n.q. – not quantified

*Coefficient of variation within the range 10-15%. Weighted average of the coefficient of variation of samples analyzed by reverse phase was under 10% in almost all the cases.

Table S2b. EVOOs minor components determined by NP-LC-DAD/FLD (mg/kg).

	Brazilian cv. Arbequina					Brazilian cv. Koroneiki					Spanish cv. Arbequina				
	Southeast		South			Southeast		South			Catalonia				
	A	B	C	D	E	B	F	C	G	H	I	J	K	L	M
**Tocopherols**															
α-Tocopherol	148	90.6	144	200*	188	196	178	190	153	254	166	172	267	163	149
β-Tocopherol	1.59	0.55*	1.30	0.57*	1.60	1.85*	1.89*	2.50	2.15	2.84	1.03*	1.22*	0.70*	1.49*	0.71*
γ-Tocopherol	1.49	n.d.	1.30	n.d.	1.90	1.52*	1.53*	4.01	3.41	3.39	1.81*	5.76	n.d.	1.60*	1.11*
δ-Tocopherol	0.35	0.63	0.74	0.24	0.53	0.96	1.03	1.00*	1.50	0.89	0.55*	0.57	0.36*	0.58	0.63
**Total tocopherols**	151	91.8	147	201	192	200	182	198	160	261	169	179	268	167	151
**Phytosterols**															
Lupeol	n.d.	n.d.	n.d.	21.4	n.d.	18.3	16.0*	n.d.	n.d.	n.d.	19.4	21.8	44.2	18.1*	13.80*
Total free sterols[a]	1179	1634	986	1739	967	495	662	400	509	773	1478	1296	1585	1331	1341
**Total phytosterols**	1179	1634	986	1760	967	514	678	400	509	773	1497	1318	1629	1349	1355
**Pigments**															
Pyropheophytin a	2.31	2.66	0.39	1.28	0.73	2.52	6.58	3.77	4.38	2.06	2.34	3.18	3.93	3.01	2.30
Pheophytin a	6.24	3.34	4.49	3.10	4.46	9.56	25.1	20.7	16.2	11.6	4.70	5.05	11.5	9.31	3.69
Pyropheophytin b	0.12*	n.d.	n.q.	n.d.	n.d.	0.24	0.47	1.02	0.77	0.36	n.d.	n.d.	0.37	0.26	n.d.
Pheophytin b	n.q.	0.28	n.q.	0.21*	n.q.	0.57	1.86	0.08	n.q.*	n.q.	0.31	0.37	0.61	0.77	0.33
Lutein	1.45	1.01	0.62	1.01	1.09	1.86	3.50	2.94	2.57	2.01	2.28	2.86	3.47	2.88	2.67
β-Carotene	4.29	4.15	2.85	2.23	4.28	6.90	12.3	9.96	8.11	8.61	3.90	4.87	7.39	4.37	3.41
**Total pigments**	14.5	11.4	8.35	7.83	10.6	21.7	49.9	38.5	32.1	24.7	13.5	16.3	27.2	20.6	12.4
**Total minor components (mg/kg)**	1785	2073	1269	2276	1341	1357	1347	865.4	1099	1218	1954	1819	2231	1954	1736

Results expressed as mean value of two independent replicates. [a]Total free sterols quantified in terms of β-Sitosterol.

*Coefficient of variation within the range 10-15%. Weighted average of the coefficient of variation of samples analyzed by reverse phase was under 5%.

Table S3. Volatile and semi-volatile compounds (µg/g) of the studied EVOOs determined by SPME-GC-MS.

EVOO samples	Hexanal	2-Hexenal	3-Hexen-1-ol	2-Hexen-1-ol	3-Ethyl-1,5-octadiene	3-Hexen-1-ol acetate	Beta ocimene	4,8-Dimethyl-1,3,7-nonatriene	Total
**Brazilian cv. Arbequina**									
Southeast A	0.53 ± 0.05^{a,d}	11.5 ± 1.11^{a}	0.49 ± 0.15^{a}	1.86 ± 0.27^{a}	n.d.	n.d.	0.24 ± 0.09^{a}	n.d.	14.6
Southeast B	0.55 ± 0.13^{a,d}	7.05 ± 0.83^{b}	n.d.	4.79 ± 1.58^{b}	0.60 ± 0.09^{a,e}	n.d.	n.d.	n.d.	13.0
South C	1.13 ± 0.39^{b,d}	15.2 ± 1.40^{c}	0.52 ± 0.04^{a}	0.20 ± 0.04^{a}	n.d.	0.48 ± 0.19^{a,d}	n.d.	0.19 ± 0.07^{a}	17.7
South D	1.18 ± 0.12^{b,d}	18.5 ± 2.26^{d}	n.d.	n.d.	0.59 ± 0.07^{a,b,e}	n.d.	0.22 ± 0.02^{a}	0.27 ± 0.02^{a,b}	20.7
South E	0.44 ± 0.00^{a}	10.6 ± 1.10^{a}	0.67 ± 0.07^{a}	0.45 ± 0.08^{a}	n.d.	n.d.	n.d.	n.d.	12.2
**Brazilian cv. Koroneiki**									
Southeast B	n.d.	1.74 ± 0.10^{e,f}	n.d.	n.d.	0.73 ± 0.09^{a,d}	n.d.	n.d.	0.43 ± 0.05^{b,c,d}	2.90
Southeast F	n.d.	1.15 ± 0.24^{e,f}	2.69 ± 0.38^{b}	n.d.	0.86 ± 0.17^{b,d,f}	n.d.	n.d.	0.36 ± 0.12^{a,d,e}	5.06
South C	n.d.	0.24 ± 0.09^{f}	5.07 ± 0.53^{c}	0.28 ± 0.08^{a}	1.87 ± 0.17^{c}	0.09 ± 0.01^{b}	n.d.	0.25 ± 0.02^{a,d}	7.80
South G	0.74 ± 0.05^{a,b,d}	3.37 ± 0.48^{e,g}	0.37 ± 0.06^{a}	n.d.	n.d.	0.83 ± 0.12^{c}	n.d.	0.47 ± 0.04^{c,e}	5.78
South H	0.76 ± 0.10 ^{a,b,d}	7.49 ± 1.73^{b}	0.56 ± 0.14^{a}	n.d.	1.08 ± 0.27^{d}	0.22 ± 0.07^{a,b,e}	n.d.	0.45 ± 0.12^{b,e}	10.6
**Spanish cv. Arbequina**									
Catalonia I	0.60 ± 0.17^{a,d}	4.00 ± 0.80^{e,g}	n.d.	1.33 ± 0.38^{a}	0.48 ± 0.09^{a,e}	0.77 ± 0.07^{c,d}	n.d.	n.d.	7.18
Catalonia J	0.43 ± 0.11^{a}	6.17 ± 0.44^{b,g}	n.d.	n.d.	0.51 ± 0.08^{a,f,e}	0.85 ± 0.10^{c}	n.d.	n.d.	7.96
Catalonia K	0.41 ± 0.09^{a}	6.12 ± 1.10^{b,g}	n.d.	n.d.	0.46 ± 0.09^{a,e}	0.52 ± 0.17^{c,d,e}	n.d.	n.d.	7.51
Catalonia L	0.90 ± 0.16^{d}	0.98 ± 0.32^{e,f}	n.d.	n.d.	0.37 ± 0.07^{a,e}	n.d.	0.18 ± 0.02^{a}	0.19 ± 0.05^{a}	2.62
Catalonia M	0.59 ± 0.16^{a,d}	3.31 ± 0.37^{e,g}	n.d.	n.d.	0.25 ± 0.03^{e}	n.d.	n.d.	n.d.	4.15

Results expressed as mean value ± standard deviation. Superscripted letters indicate significant differences between samples ($p < 0.05$; One-way ANOVA followed by Tukey's post hoc test). n.d. – not detected.

Table S4a: Identification parameters of minor components determined by RP-LC-MS in the studied samples.

Compound	Molecular formula	Structure	Retention Time (min)	Theoretical *m/z*	Error (ppm)
**Simple phenols, phenolic acids and related substances**					
Quinic acid	$C_7H_{12}O_6$	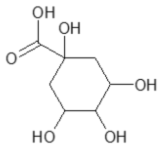	0.9	191.0561	1.1
Oxidized hydroxytyrosol	$C_8H_8O_3$	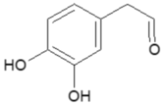	1.1	151.0401	-3.3
Hydroxytyrosol	$C_8H_{10}O_3$	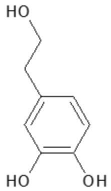	1.8	153.0557	-0.6
Eudesmic acid	$C_{10}H_{12}O_5$	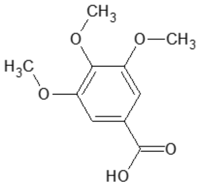	2.1	211.0612	-0.5

Compound	Molecular formula	Structure	Retention Time (min)	Theoretical *m/z*	Error (ppm)
**Simple phenols, phenolic acids and related substances (cont.)**					
Tyrosol	$C_8H_{10}O_2$	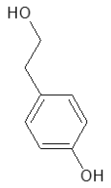	2.6	137.0608	0.0
Vanillic acid	$C_8H_8O_4$	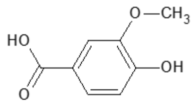	3.0	167.0350	0.0
p-Coumaric acid	$C_9H_8O_3$	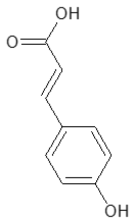	3.6	163.0401	-2.4
Vanillin	$C_8H_8O_3$	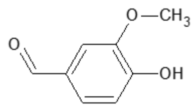	3.9	151.0401	-1.3

Compound	Molecular formula	Structure	Retention Time (min)	Theoretical *m/z*	Error (ppm)
**Simple phenols, phenolic acids and related substances (cont.)**					
Ferulic acid	$C_{10}H_{10}O_4$	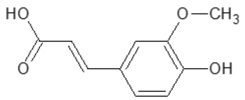	4.0	193.0506	-1.0
Hydroxytyrosol acetate	$C_{10}H_{12}O_4$	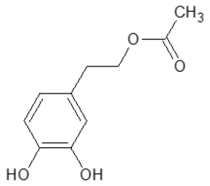	4.5	195.0663	0.0
**Secoiridoids and derivatives**					
Decarboxy-methyl-elenolic acid	$C_9H_{12}O_4$	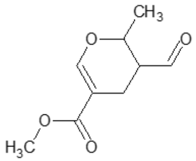	3.4	183.0663	0.0
Desoxy-elenolic acid	$C_{11}H_{14}O_5$	*	4.1	225.0768	0.4
Hydroxy-elenolic acid	$C_{11}H_{14}O_7$	*	4.2	257.0667	0.0

Compound	Molecular formula	Structure	Retention Time (min)	Theoretical *m/z*	Error (ppm)
**Secoiridoids and derivatives (cont.)**					
Elenolic acid	$C_{11}H_{14}O_6$	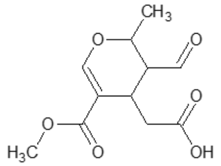	4.8	241.0718	-0.8
Hydroxy-decarboxy-methyl-oleuropein aglycone	$C_{17}H_{20}O_7$	*	4.9	335.1136	0.6
Decarboxy-methyl-oleuropein aglycone (isomer 1)	$C_{17}H_{20}O_6$	*	5.5	319.1187	0.6
Decarboxy-methyl-oleuropein aglycone (isomer 2)	$C_{17}H_{20}O_6$	*	6.1	319.1187	0.0
Oleuropein aglycone (isomer 1)	$C_{19}H_{22}O_8$	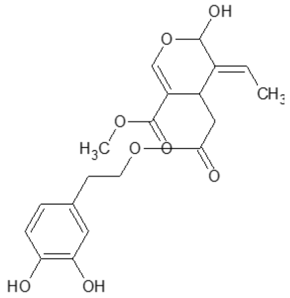	6.3	377.1242	-0.5
Oleuropein aglycone (isomer 2)	$C_{19}H_{22}O_8$		6.7	377.1242	0.0
Oleuropein aglycone (isomer 3)	$C_{19}H_{22}O_8$		8.4	377.1242	-0.5
Oleuropein aglycone (isomer 4)	$C_{19}H_{22}O_8$		9.0	377.1242	-0.8
Oleuropein aglycone (isomer 5)	$C_{19}H_{22}O_8$		11.1	377.1242	1.6

Compound	Molecular formula	Structure	Retention Time (min)	Theoretical *m/z*	Error (ppm)
**Secoiridoids and derivatives (cont.)**					
10-Hydroxyoleuropein aglycone (isomer 1)	$C_{19}H_{22}O_9$	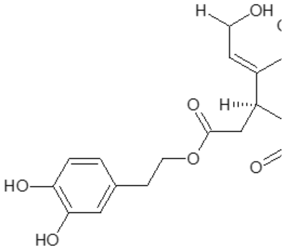	6.5	393.1191	0.0
10-Hydroxyoleuropein aglycone (isomer 2)	$C_{19}H_{22}O_9$		6.7	393.1191	-1.3
10-Hydroxyoleuropein aglycone (isomer 3)	$C_{19}H_{22}O_9$		6.8	393.1191	-1.0
Decarboxymethyl-ligstroside aglycone	$C_{17}H_{20}O_5$	*	7.1	303.1238	0.0
Ligstroside aglycone (isomer 1)	$C_{19}H_{22}O_7$	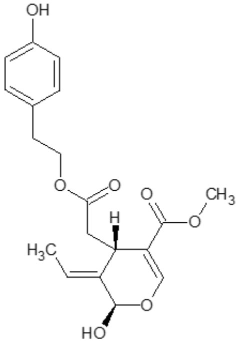	7.7	361.1293	0.5
Ligstroside aglycone (isomer 2)	$C_{19}H_{22}O_7$		8.3	361.1293	-1.1
Ligstroside aglycone (isomer 3)	$C_{19}H_{22}O_7$		10.2	361.1293	0.0
Ligstroside aglycone (isomer 4)	$C_{19}H_{22}O_7$		10.7	361.1293	-0.3
Ligstroside aglycone (isomer 5)	$C_{19}H_{22}O_7$		11.1	361.1293	1.7
Methyl-decarboxymethyl-oleuropein aglycone (isomer 1)	$C_{18}H_{22}O_6$	*	8.1	333.1344	-0.9

Compound	Molecular formula	Structure	Retention Time (min)	Theoretical *m/z*	Error (ppm)
**Secoiridoids and derivatives (cont.)**					
Methyl-decarboxymethyl-oleuropein aglycone (isomer 2)	$C_{18}H_{22}O_6$	*	8.4	333.1344	0.0
Dehydro-oleuropein aglycone	$C_{19}H_{20}O_8$	*	8.3	375.1085	0.8
Methyl-oleuropein aglycone (isomer 1)	$C_{20}H_{24}O_8$	*	8.6	391.1398	-0.5
Methyl-oleuropein aglycone (isomer 2)	$C_{20}H_{24}O_8$	*	9.0	391.1398	-1.3
Dehydro-ligstroside aglycone	$C_{19}H_{20}O_7$	*	10.1	359.1136	-1.7
**Flavonoids**					
Luteolin	$C_{15}H_{10}O_6$	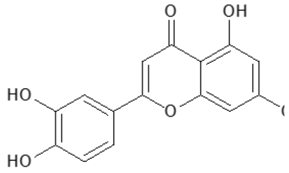	5.7	285.0405	-1.0

Compound	Molecular formula	Structure	Retention Time (min)	Theoretical *m/z*	Error (ppm)
**Flavonoids (cont.)**					
Apigenin	$C_{15}H_{10}O_5$	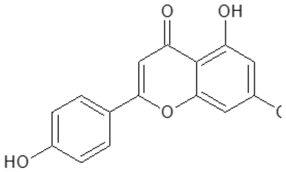	7.0	269.0455	-0.7
Naringenin	$C_{15}H_{12}O_5$	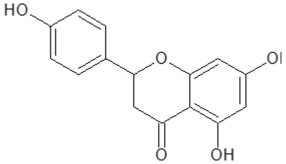	7.1	271.0612	0.0
Diosmetin	$C_{16}H_{12}O_6$	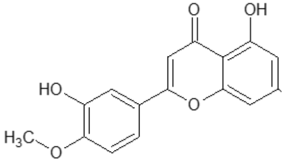	7.4	299.0561	-0.3

Compound	Molecular formula	Structure	Retention Time (min)	Theoretical *m/z*	Error (ppm)
**Lignans**					
Syringaresinol	$C_{22}H_{26}O_8$	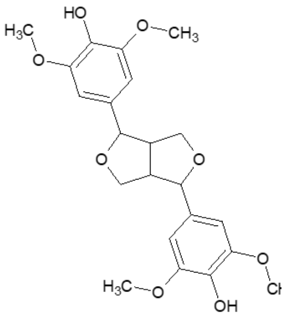	6.1	417.1555	-1.7
Pinoresinol	$C_{20}H_{22}O_6$	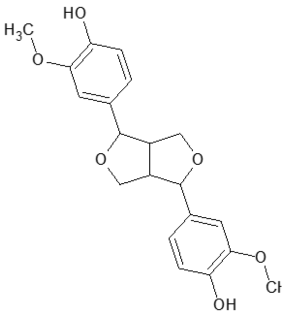	6.5	357.1344	-1.4

Compound	Molecular formula	Structure	Retention Time (min)	Theoretical *m/z*	Error (ppm)
**Lignans (cont.)**					
Acetoxypinoresinol	$C_{22}H_{24}O_8$	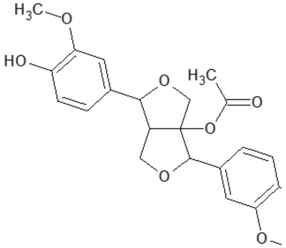	6.8	415.1398	1.2
**Triterpenic compounds**					
Maslinic acid	$C_{30}H_{48}O_4$	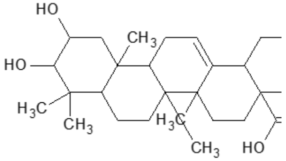	18.5	471.3480	1.1
Betulinic acid	$C_{30}H_{48}O_3$	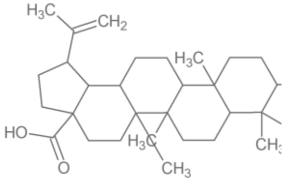	21.2	455.3531	0.2
Oleanolic acid	$C_{30}H_{48}O_3$	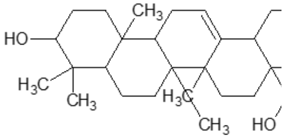	21.5	455.3531	-0.4

Compound	Molecular formula	Structure	Retention Time (min)	Theoretical *m/z*	Error (ppm)
**Free fatty acids**					
Linolenic acid	$C_{18}H_{30}O_2$	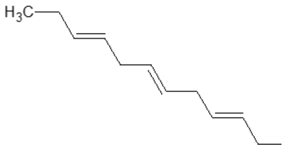	21.3	277.2173	0.0
Palmitoleic acid	$C_{16}H_{30}O_2$	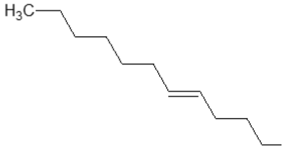	21.9	253.2173	-0.8
Linoleic acid	$C_{18}H_{32}O_2$	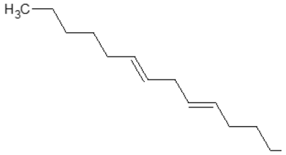	22.3	279.2330	-0.7

*Missing structures due to the possibility of more than one isomer structure conformation. Proposed identification matches previous studies [26-27].

Table S4b: Identification parameters of minor components determined by NP-LC-DAD/FLD in the studied samples.

Compound	Molecular formula	Structure	Retention Time (min)	Wavelength (nm)
**Tocopherols**				
α-Tocopherol	$C_{29}H_{50}O_2$	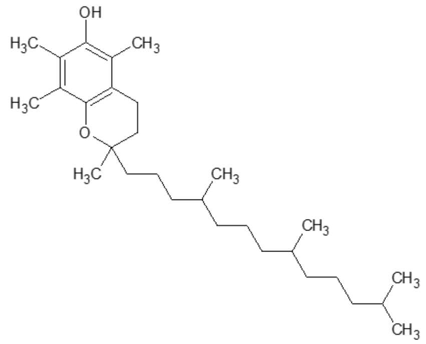	1.9	
				$\lambda_{excitation}$= 295; $\lambda_{emission}$= 330
β-Tocopherol	$C_{28}H_{48}O_2$	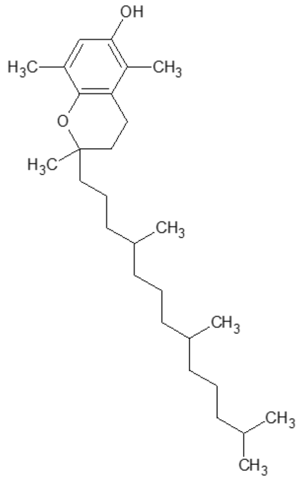	2.5	

Compound	Molecular formula	Structure	Retention Time (min)	Wavelength (nm)
**Tocopherols (cont.)**				
γ-Tocopherol	$C_{28}H_{48}O_2$	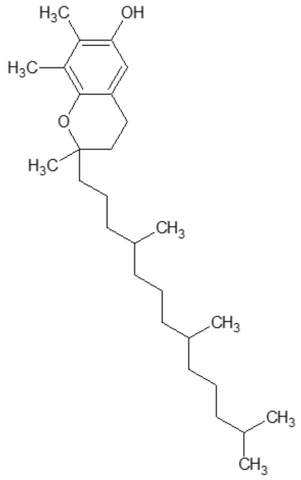	2.7	$\lambda_{excitation}$= 295; $\lambda_{emission}$= 330
δ-Tocopherol	$C_{27}H_{46}O_2$	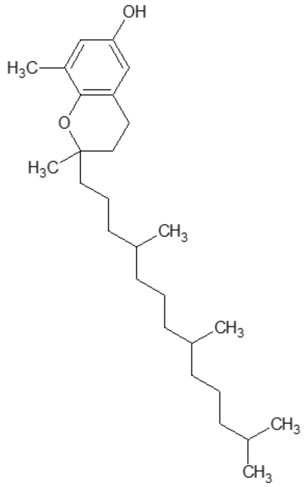	3.8	

Compound	Molecular formula	Structure	Retention Time (min)	Wavelength (nm)
**Phytoesterols**				
Lupeol	$C_{30}H_{50}O$	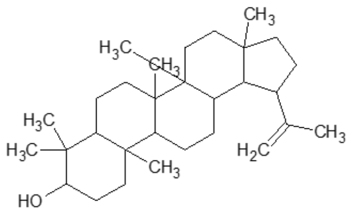	3.3	λ= 210
β-Sitosterol (coeluting with other free sterols)	$C_{29}H_{50}O$	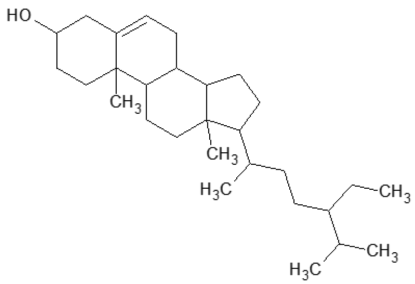	6.3	
**Pigments**				

β-Carotene	$C_{40}H_{56}$	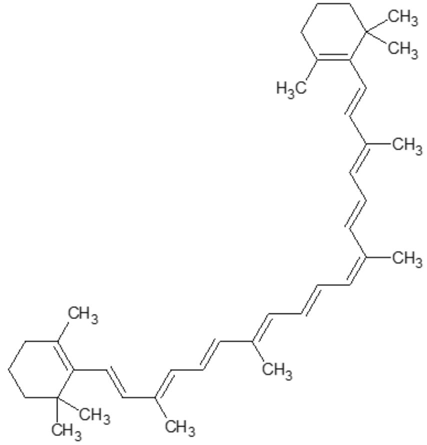	1.1	λ= 454

Compound	Molecular formula	Structure	Retention Time (min)	Wavelength (nm)
**Pigments (cont.)**				
Pyropheophytin a	$C_{53}H_{72}N_4O_3$	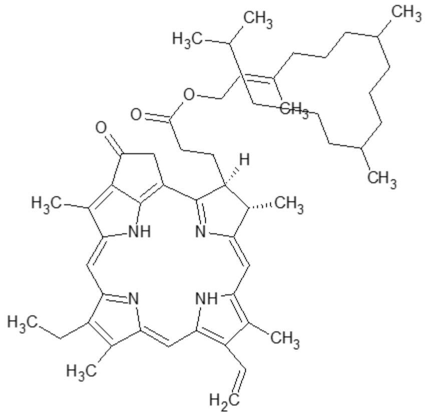	2.9	λ= 409[a]
Pheophytin a	$C_{55}H_{74}N_4O_5$	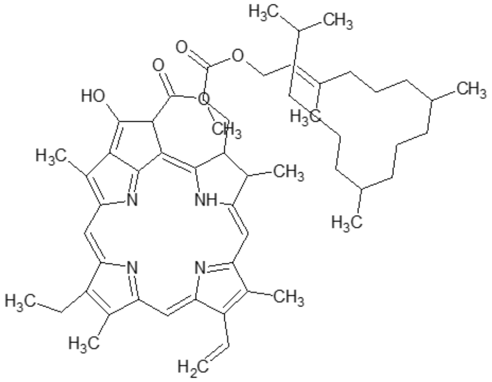	3.4	λ= 409 [a]

Compound	Molecular formula	Structure	Retention Time (min)	Wavelength (nm)
**Pigments (cont.)**				
Pyropheophytin b	$C_{53}H_{72}N_4O_3$	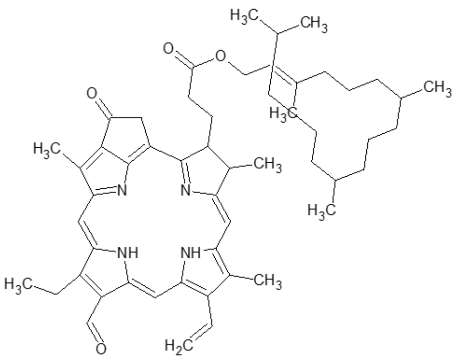	5.1	λ= 430 [a]
Pheophytin b	$C_{55}H_{72}N_4O_6$	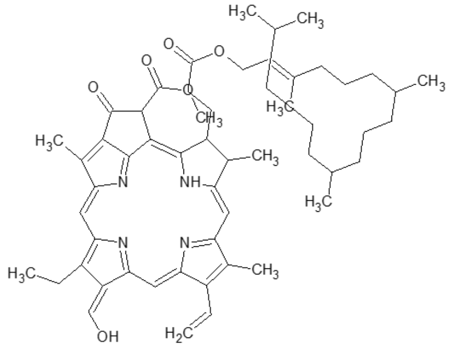	5.9	λ= 430 [a]

Compound	Molecular formula	Structure	Retention Time (min)	Wavelength (nm)
**Pigments (cont.)**				
Lutein	$C_{40}H_{56}O_2$	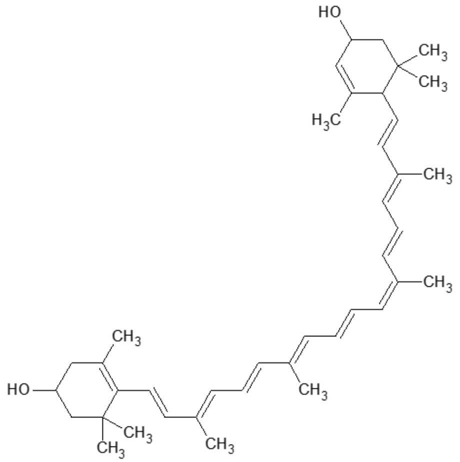	16.5	λ= 446

[a] Fluorescence $\lambda_{excitation}$= 430 nm and $\lambda_{emission}$= 660 nm were also used starting at 4.3 min to assist on compounds identification.

Table S5a: Analytical parameters of the RP-LC-MS method.

Compound	Equation[1]	Compound used for quantification	r[2]	LOD (µg/mL)	LOQ (µg/mL)	Concentration range (µg/mL) for each calibration. curve[2]
**Simple phenols, phenolic acids, and related substances**						
Quinic acid	y1 = -1475.5x + 56036.2<br>y2 = 556532.7x + 31086.2	Quinic acid	0.9965<br>0.9831	0.025	0.082	1 = 0.5 – 19.0<br>2 = 19.0 – 95.0

Oxidized Hydroxytyrosol<br>*Hydroxytyrosol acetate*<br>*Hydroxytyrosol*	y1 = -5206.2x + 78295.4<br>y2 = 30642.3x + 74026.9	Hydroxytyrosol	0.9847<br>0.9847	0.032	0.108	1 = 0.1 – 5.5<br>2 = 2.8 – 27.5
Eudesmic acid<br>*Vanillic acid*	y = 3063.9x + 11900	Vanillic acid	0.9872	0.069	0.230	0.1 – 12.5
Tyrosol	y = 6366.07 + 28675.1	Tyrosol	0.9907	0.036	0.121	0.1 – 12.5
p-Coumaric acid	y1 = -105.7x + 17919.5	*p*-Coumaric acid	0.9964	0.053	0.177	0.3 – 11.0
Vanillin	y1= 4902.1x + 21391.1<br>y2 = 87201.8x + 9830.8	Vanillin	0.9920<br>0.9815	0.018	0.061	1 = 0.2 – 5.0<br>2 = 5.0 – 50.0
Ferulic acid	y = 698.1x + 14835	Ferulic acid	0.9942	0.069	0.229	0.3 – 11.0
**Secoiridoids and derivatives**						
All compounds	y1= 207591x + 378525<br>y2 = 2046776x + 196978.8<br>y3= 6846775.2x + 91535.2	Oleuropein	0.9930<br>0.9942<br>0.9905	0.005	0.018	1 = 1.3 – 6.5<br>2 = 6.5 – 52.0<br>3 = 52.0 – 260

Compound	Equation[1]	Compound used for quantification	r^2	LOD (µg/mL)	LOQ (µg/mL)	Concentration range (µg/mL) for each calibration. curve[2]
**Flavonoids**						
Luteolin	y1 = 13739.8x + 341457	Luteolin	0.9820	0.002	0.005	1 = 0.1 – 2.8
Diosmetin	y2 = 582522.2x + 184363.2		0.9855			2 = 2.8 – 27.5
Apigenin	y = 51761.1x + 346578	Apigenin	0.9902	0.003	0.010	0.1 – 5.00
Naringenin						
**Lignans**						
Syringaresinol	y1 = 17539.3x + 191180	Pinoresinol	0.9908	0.007	0.022	1 = 0.3 – 6.5
Pinoresinol	y2 = 653595.9x + 122757.7		0.9901			2 = 6.5 – 65.0
Acetoxypinoresinol						
**Triterpenic compounds**						
Maslinic acid	y1 = 423427x + 954049	Maslinic acid	0.9814	0.001	0.003	1 = 0.5 – 5.0
	y2 = 3207604x + 410142.6		0.9909			2 = 5.0 – 20.0
	y3 = 8859417x + 152689		0.9805			3 = 20.0 - 100
Betulinic acid	y1 = -65988x + 233392	Betulinic acid	0.9863	0.010	0.035	1 = 0.5 – 5.0
	y2 = 455365.1x + 136781.9		0.9855			2 = 5.0 – 20.0
	y3 = 2052976.7x + 63314.7		0.9851			3 = 20.0 - 100
Oleanolic acid	y1 = 118738x + 3244683	Oleanolic acid	0.9811	0.0004	0.002	1 = 0.1 – 1.2
	y2=2297590x + 1553599.9		0.9938			2 = 1.2 – 5.0
	y3 = 8027706.1x + 442800		0.9817			3 = 5.0 – 25.0

Compound	Equation[1]	Compound used for quantification	r^2	LOD (µg/mL)	LOQ (µg/mL)	Concentration range (µg/mL) for each calibration. curve[2]
**Free fatty acids**						
	y1 = 524403x + 5581010		0.9884			1 = 0.1 – 1.4
Linolenic acid	y2 = 4066140x + 2816839.7	Linolenic acid	0.9998	0.0001	0.0004	2 = 1.4 – 5.5
	y3 = 14432203x + 1051767		0.9943			3 = 5.5 – 27.5
	y1 = 145890x + 604729		0.9896			1 = 0.5 – 5.0
Palmitoleic acid	y2 = 1811226x + 284840.8	Palmitoleic acid	0.9861	0.001	0.003	2 = 5.0 – 20.0
	y3 = 5693458.3x + 105003		0.9811			3 = 20.0 - 100
	y1 = 120803x + 551305		0.9947			1 = 0.8 – 8.5
Linoleic acid	y2 = 1731694x + 356473.9	Linoleic acid	0.9998	0.003	0.011	2 = 8.5 – 34.0
	y3 = 12526120x + 71130.6		0.99762			3 = 34.0 - 170

[1] y1, y2 or y3 are the equations for the different calibration ranges established (1, 2 or 3, respectively), according to the compound concentration ([2]).

Table S5b: Analytical parameters of the NP-LC-DAD/FLD method.

Compound	Equation[1]	Compound used for quantification	r^2	LOD (µg/mL)	LOQ (µg/mL)	Concentration range (µg/mL) for each calibration. curve[2]
**Tocopherols**						
α-Tocopherol	y1 = 0.256x + 0.192	α-Tocopherol	0.9899	0.096	0.319	1 = 0.06 – 7.5
	y2 = 1.785x + 0.160		0.9834			2 = 7.5 – 41.4

Compound	Equation[1]	Compound used for quantification	r2	LOD (µg/mL)	LOQ (µg/mL)	Concentration range (µg/mL) for each calibration. curve[2]
**Tocopherols (cont.)**						
β-Tocopherol	y1 = 0.019x + 0.044<br>y2 = 0.436x + 0.034	β-Tocopherol	0.9939<br>0.9830	0.013	0.044	1 = 0.01 – 1.2<br>2 = 1.2 – 10.0
γ-Tocopherol	y1 = 0.007x + 0.050<br>y2 = 0.243x + 0.035	γ-Tocopherol	0.9837<br>0.9925	0.014	0.046	1 = 0.01 – 0.5<br>2 = 0.5 – 10.0
δ-Tocopherol	y1 = -0.005x + 0.026<br>y2 = 0.200x + 0.022	δ-Tocopherol	0.9989<br>0.9977	0.006	0.020	1 = 0.01 – 1.2<br>2 = 1.2 – 7.5
**Phytoesterols**						
Lupeol	y1 = -0.054x + 0.194<br>y2= 0.280x + 0.203	Lupeol	0.9950<br>0.9826	0.999	3.329	1 = 0.1 – 6.9<br>2 = 6.9 – 55.0
β-Sitosterol (coeluting with other free sterols)	y1 = -0.322x + 0.310<br>y2 = 3.792x + 0.300	β-Sitosterol	0.9988<br>0.9834	3.640	12.135	1 = 1.0 – 50.0<br>2 = 50.0 – 400
**Pigments**						
β-Carotene	y1= -0.002x + 0.019<br>y2 = -1.870x + 54.46	β-Carotene	1.0000<br>0.9842	0.006	0.018	1 = 0.01 – 1.2<br>2 = 1.2 – 10.0
Pyropheophytin a<br>*Pheophytin a*	y1 = 0.037x + 0.020<br>y2 = 1.277x + 0.018	Chlorophyll a	0.9996<br>0.9878	0.025	0.082	1 = 0.05 – 6.2<br>2 = 6.2 – 50.0
Pyropheophytin b<br>*Pheophytin b*	y1 = 0.029x + 0.016<br>y2 = 0.319x + 0.014	Chlorophyll b	0.9989<br>0.9894	0.024	0.080	1 = 0.03 – 1.2<br>2 = 1.2 – 10.0

Lutein	y1 = -0.0100 + 0.010<br>y2= 0.100x + 0.010	Lutein	0.9990<br>0.9876	0.004	0.012	1 = 0.01 – 1.2<br>2 = 1.2 – 10.0

[1] y1, y2 or y3 are the equations for the different calibration ranges established (1, 2 or 3, respectively), according to the compound concentration ([2]).

Table S6: Volatile compounds detected in the studied EVOOs determined by SPME-GC-MS, including identification parameters and related aroma notes.

Volatile compounds	Aroma notes	LRI_{lit}	LRI_{exp} ($Error_{LRI}$; %)	Similarity Index (SI)
**Aldehydes**				
Hexanal	Grass, tallow, fat[1]	780	777 (0.4)	61
2-Hexenal	Apple, green[1]	850	837 (1.5)	95
**Alcohols**				
3-Hexen-1-ol	Green, fruity[1]	858	841 (2.0)	81
2-Hexen-1-ol	Leaf, green[1]	887	856 (3.5)	87
**Hydrocarbons**				
3-Ethyl-1,5-octadiene	-	-	992 (-)	80
4,8-Dimethyl-1,3,7-nonatriene	-	1114	1114 (0)	83
Beta ocimene	Sweet, herb[1]	1050	1045 (0.5)	92
**Ester**				
3-Hexen-1-ol, acetate	Banana and walnut husk[1]	1009	993 (1.6)	86

LRI_{lit}: Linear retention index from the literature (NIST WebBook; accessed on: https://webbook.nist.gov/chemistry/. LRI_{exp}: Mean experimental linear retention index. Volatile compounds were identified by the following criteria: (SI) >60% and $Error_{LRI}$< 0.5%; or SI > 80%. [1]Aroma notes described on Flavornet database (accessed on: http://www.flavornet.org/flavornet.html).
